# Uncovering the burden of hidden ciliopathies in the 100 000 Genomes Project: a reverse phenotyping approach

**DOI:** 10.1136/jmedgenet-2022-108476

**Published:** 2022-06-28

**Authors:** Sunayna Best, Jing Yu, Jenny Lord, Matthew Roche, Christopher Mark Watson, Roel P J Bevers, Alex Stuckey, Savita Madhusudhan, Rosalyn Jewell, Sanjay M Sisodiya, Siying Lin, Stephen Turner, Hannah Robinson, Joseph S Leslie, Emma Baple, Genomics England Research Consortium, Carmel Toomes, Chris Inglehearn, Gabrielle Wheway, Colin A Johnson

**Affiliations:** 1 Division of Molecular Medicine, Leeds Institute of Medical Research, University of Leeds, Leeds, UK; 2 Yorkshire Regional Genetics Service, Leeds Teaching Hospitals NHS Trust, Leeds, UK; 3 Nuffield Department of Clinical Neurosciences, John Radcliffe Hospital, University of Oxford, Oxford, UK; 4 University Hospital Southampton NHS Foundation Trust, Southampton, UK; 5 Faculty of Medicine, Human Development and Health, University of Southampton, Southampton, UK; 6 Windsor House Group Practice, Mid Yorkshire Hospitals NHS Trust, Leeds, UK; 7 North East and Yorkshire Genomic Laboratory Hub, Central Lab, Leeds Teaching Hospitals NHS Trust, Leeds, UK; 8 Genomics England, Queen Mary University of London, London, UK; 9 St. Paul’s Eye Unit, Royal Liverpool University Hospital, Liverpool, UK; 10 University College London (UCL) Queen Square Institute of Neurology, London, UK; 11 Chalfont Centre for Epilepsy, Chalfont, UK; 12 Department of Ophthalmology, Torbay and South Devon NHS Foundation Trust, Torquay, UK; 13 Exeter Genomics Laboratory, Royal Devon and Exeter NHS Foundation Trust, Exeter, UK; 14 RILD Wellcome Wolfson Centre, University of Exeter Medical School, Exeter, UK; 15 Peninsula Clinical Genetics Service, Royal Devon and Exeter NHS Foundation Trust, Exeter, UK

**Keywords:** genomics, genetics, medical

## Abstract

**Background:**

The 100 000 Genomes Project (100K) recruited National Health Service patients with eligible rare diseases and cancer between 2016 and 2018. PanelApp virtual gene panels were applied to whole genome sequencing data according to Human Phenotyping Ontology (HPO) terms entered by recruiting clinicians to guide focused analysis.

**Methods:**

We developed a reverse phenotyping strategy to identify 100K participants with pathogenic variants in nine prioritised disease genes (*BBS1, BBS10, ALMS1, OFD1, DYNC2H1, WDR34, NPHP1, TMEM67, CEP290*), representative of the full phenotypic spectrum of multisystemic primary ciliopathies. We mapped genotype data ‘backwards’ onto available clinical data to assess potential matches against phenotypes. Participants with novel molecular diagnoses and key clinical features compatible with the identified disease gene were reported to recruiting clinicians.

**Results:**

We identified 62 reportable molecular diagnoses with variants in these nine ciliopathy genes. Forty-four have been reported by 100K, 5 were previously unreported and 13 are new diagnoses. We identified 11 participants with unreportable, novel molecular diagnoses, who lacked key clinical features to justify reporting to recruiting clinicians. Two participants had likely pathogenic structural variants and one a deep intronic predicted splice variant. These variants would not be prioritised for review by standard 100K diagnostic pipelines.

**Conclusion:**

Reverse phenotyping improves the rate of successful molecular diagnosis for unsolved 100K participants with primary ciliopathies. Previous analyses likely missed these diagnoses because incomplete HPO term entry led to incorrect gene panel choice, meaning that pathogenic variants were not prioritised. Better phenotyping data are therefore essential for accurate variant interpretation and improved patient benefit.

**What is already known on this topic**

- Whole genome sequencing and targeted gene-panel analysis have improved molecular diagnosis rates for patients with multisystemic ciliopathies.

**What this study adds**

- Reverse phenotyping from 100 00 Genomes Project data has identified 62 reportable molecular diagnoses with variants in nine prioritised ciliopathy genes, of which 18 are new diagnoses not reported by Genomics England Ltd.
- Furthermore, we identified 11 unreportable molecular diagnoses in these genes, but these lacked adequate clinical data to justify returning the findings to recruiting clinicians.

**How this study might affect research, practice and/or policy**

- Reverse phenotyping can improve molecular diagnosis rates from large-scale genomic projects.
- Comprehensive phenotypic data are essential to facilitate accurate variant interpretation.

## Introduction

The 100 000 Genomes Project (100K) is a combined diagnostic and research initiative managed by Genomics England Ltd (GEL). It aimed to sequence 100 000 genomes from 70 000 participants seen within the UK National Health Service (NHS) with either selected rare diseases or cancers, the latter allowing comparison of matched germline and somatic tumour genomes.^1 2^ To take part in 100K, participants consented to receive a result ‘relevant to the explanation, main diagnosis or treatment of the disease for which the patient was selected for testing’ (the ‘pertinent finding’), if identified.^3^ Furthermore, they consented to allow access to their fully anonymised genome sequence data and phenotype information for approved academic and commercial researchers. Short-read genome sequencing was performed using Illumina ‘TruSeq’ library preparation kits for read lengths 100 bp and 125 bp (Illumina HiSeq 2500 instruments), or 150 bp reads (HiSeq X). These generated a mean read depth of 32× (range, 27–54) and a depth >15× for at least 95% of the reference human genome.^2^ In the Main Programme Data Release 12 (5 June 2021) used in this study, data were available for 88 844 individuals: 71 597 in the rare diseases arm (33 208 probands and 33 388 relatives) and 17 247 in the cancer arm.

Large-scale genomic studies such as the 100K offer the opportunity to perform reverse phenotyping for genes of interest. In traditional forward genetics, observation of clinical features prompts differential diagnoses and the subsequent evaluation of genes with potentially pathogenic variants (phenotype-to-genotype model). In reverse phenotyping, the search begins with the identification of potentially pathogenic variants, which are then mapped in a reverse strategy against the key clinical features of patients in order to guide phenotyping. Patients with potential causative variants in the selected genes are assessed to see if their clinical features match the associated disease phenotype and inheritance pattern reported in the medical literature (genotype-to-phenotype model).

Reverse phenotyping strategies have been especially successful for diseases characterised by high heterogeneity and complex phenotypes. For example, reverse phenotyping is helping to uncover the genetic architecture of pulmonary arterial hypertension.^4^ Reverse phenotyping allowed diagnosis of 18/64 previously unsolved patients with steroid-resistant nephrotic syndrome through analysis of 298 causative genes after whole exome sequencing (WES). This was followed by multidisciplinary team (MDT) discussion and recommended additional examinations to detect previously overlooked signs or symptoms of the syndromic genetic disorder that was guided by knowledge of the identified pathogenic variants.^5^ Reverse phenotyping also provides an opportunity to extend or refine the phenotype for disease-associated genes, as demonstrated for a family with an *INPP5E*-related ciliopathy.^6^

Ciliopathies are a group of rare inherited disorders caused by abnormalities of structure or function of primary cilia (the ‘cell’s antenna’)^7^ or motile cilia (organelles responsible for the movement of fluid over the surface of cells).^8 9^ Ciliopathy syndromes present as a clinical spectrum, ranging from relatively common single-system disorders such as retinal or renal ciliopathies, through to rare, complex, multisystem syndromes. There is considerable phenotypic and genetic heterogeneity between the >35 reported ciliopathy syndromes.^9 10^ Common, shared clinical features include renal malformations and/or renal dysfunction, retinal dystrophy, developmental delay, intellectual disability, cerebellar abnormalities, obesity and skeletal abnormalities.^11^ Collectively, ciliopathies are thought to affect up to 1 in 2000 people based on three common frequent clinical features: renal cysts (1 in 500 adults), retinal degeneration (1 in 3000) and polydactyly (1 in 500).^12^ Multisystemic ciliopathies can be grouped into metabolic/obesity ciliopathies, neurodevelopmental ciliopathies and skeletal ciliopathies. The variety in systems involvement reflects the critical role of cilia in development and health.^2^

We recently published a study determining a research molecular diagnosis for n=43/83 (51.8%) of probands recruited under primary ciliopathy categories by GEL, comprising the ‘Congenital Malformations caused by Ciliopathies’ cohort.^13^ We noted that a high proportion of diagnoses were caused by variants in non-ciliopathy disease genes (n=19/43, 44.2%). We hypothesised that this reflects difficulties in the clinical recognition of ciliopathies, as well as practical challenges in recruiting participants to 100K under appropriate rare disease domains. It is therefore reasonable to assume that there are also ‘hidden’ patients with ciliopathies recruited to alternative categories.

## Methods

In order to improve the rate of successful molecular diagnosis for unsolved 100K participants with known or suspected ciliopathies, we developed a reverse phenotyping strategy for selected exemplar genes that are most frequently mutated as a cause of primary multisystemic ciliopathies.

### Selection of common multisystemic ciliopathy genes to assess

A literature review was undertaken to determine the most common genetic causes of multisystemic primary ciliopathies: Bardet-Biedl syndrome (BBS) and Alström syndrome (metabolic/obesity ciliopathies); Joubert syndrome (JBTS), Meckel-Gruber syndrome (MKS) and orofaciodigital syndrome (OFD) (neurodevelopmental ciliopathies); the skeletal ciliopathy Jeune asphyxiating thoracic dystrophy (JATD) and nephronophthisis (isolated or syndromic renal ciliopathy).^2^ Disease genes causative of ≥10% of the total syndrome burden were selected for inclusion in the reverse phenotyping analysis and are summarised alongside referenced literature (online supplemental table 1). Where disease genes are known to cause multiple ciliopathy syndromes, all associated conditions are included in the table. On this basis, nine disease genes were selected as exemplars that span the extensive phenotypic range of primary multisystemic ciliopathies: *BBS1, BBS10, ALMS1, OFD1, DYNC2H1, WDR34, NPHP1, TMEM67* and *CEP290*. All have autosomal recessive inheritance except *OFD1* which is associated with X linked dominant OFD type 1 (OFD-1) and X linked recessive JBTS.^13^ Almost all individuals with OFD-1 are female; the few affected males are reported to be malformed fetuses delivered by an affected female.

10.1136/jmedgenet-2022-108476.supp1 Supplementary data

### Identification of solved participants with causative variants in representative ciliopathy disease genes

All analysis on the GEL datasets were performed within a secure workspace called the ‘Research Environment’. Clinical and participant data were integrated and analysed using ‘LabKey’ data management software. Previously reported diagnoses were identified using data in the NHS Genomics Medical Centres (GMC) ‘Exit Questionnaire’. The Exit Questionnaire is completed by the clinicians at the GMC for each closed case, and summarises the extent to which a participant’s diagnosis can be explained by the combined variants reported to the GMC from GEL and clinical interpretation providers. Data in Exit Questionnaires were filtered for reports containing variants in the nine ciliopathy disease genes, where the ‘case solved family’ was annotated as ‘yes’ (solved) or ‘partially’ (partially solved).

### Selection of key clinical terms associated with selected ciliopathy genes

A literature search of review articles prioritised the key clinical terms for each of the nine selected ciliopathy genes. This assessed the potential match against phenotype and justification for reporting new molecular findings. Approved researchers submit a ‘Researcher Identified Diagnosis’ (RID) form using the secure GEL ‘Airlock’ system. This is then sent to the participant’s recruiting clinician for consideration of the fit to phenotype and the interpretation of variant pathogenicity, followed by decisions about whether the finding should be reported back to the participant. Usually, such cases are discussed at multiMDT meetings involving clinical scientists, researchers and clinicians. Variants classed as likely pathogenic or pathogenic and felt to be a good clinical match for phenotype, must be molecularly confirmed and formally reported by an NHS-accredited diagnostic laboratory before being fed back to the participant by the clinician responsible for their care.^3^ Decisions about feedback of variants of uncertain clinical significance (VUS) to participants are the responsibility of individual clinicians following MDT discussion, but are usually not fed back.

The rationale for selection of key features is presented in table 1, supported by key references from the literature. To allow easier categorisation and to protect participant anonymity, they are grouped into 11 body systems. Without specific participant consent for research studies, we are unable to present clinical features that would potentially identify individuals to within five participants in 100K.^3^ Major features (M) are those present in >50% of affected individuals and/or listed as major diagnostic or characteristic features in the cited literature. Minor features (m) are those present in <50% of affected individuals and/or listed as minor diagnostic features. The EMBL-EBI Ontology Lookup Service was used to supplement linked Human Phenotyping Ontology (HPO) terms for each key clinical term, to facilitate capture of a wider selection of appropriate HPO terms that were entered by recruiting clinicians (available from https://www.ebi.ac.uk/ols/index). The list of acceptable linked HPO terms is available in online supplemental table 2.

**Table 1:** Key clinical features for ciliopathy syndromes associated with the nine selected ciliopathy genes of interest

System	Ciliopathy syndrome	BBS	ALMS	JATD	OFD-1	Nephronophthisis	JBTS	MKS	LCA/EOSRD
	Reference(s)	40	30	41	13 39	42	43	44	45
	Chosen ciliopathy gene(s) associated with syndrome	*BBS1, BBS10, TMEM67, CEP290*	*ALMS1*	*DYNC2H1, WDR34*	*OFD1*	*NPHP1* (isolated+syndromic), *TMEM67+CEP290* (syndromic)	*TMEM67, CEP290, NPHP1, OFD1*	*TMEM67, CEP290*	*CEP290*
Ophthalmic	Retinal dystrophy	**M**	**M**	**M**		m*†‡	m*†‡		**M**
	Abnormality of eye movement					m*†‡	**M**		**M**
	Lens opacities								**M**
	Keratoconus								**M**
Gastrointestinal	Abnormality of the liver		**m**	**M**	**m**	m*†‡	m*†‡	**M**	
	Abnormality of the gut	**m**		**m**					
Renal	Abnormal renal morphology/dysfunction	**M**	**M**	**M**	**M**	**M**	m*†	**M**	
Genitourinary	Abnormality of the genitourinary system	**M**	**m**					**m**	
Cardiovascular	Cardiomyopathy		**M**						
	Laterality defect	**m**				m*†	m*†	**m**	
	Congenital heart disease	**m**		**m**				**m**	
	Hypertension		**m**						
Sensory	SNHL	**m**	**M**						
	Glue ear		**m**						
	Chronic otitis media		**m**		**m**				
	Abnormality of the sense of smell	**M**							
Endocrine/Metabolic	Hypogonadotrophic hypogonadism	**M**	**M**						
	Glucose intolerance		**M**						
	Obesity	**M**	**M**						
	Hypertriglyceridemia		**M**						
	Thyroid abnormality	**m**	**m**					**m**	
	Polycystic ovarian syndrome	**m**			**m**				
Neurological	Intellectual disability	**M**	**m**		**M**	m*†	**M**		
	Neurodevelopmental delay	**M**	**m**				**M**		
	Hypotonia		**m**				**M**		
	Ataxia		**m**				**M**		
	Abnormality of brain morphology			**m**	**M**	m*†	**M**	**M**	
	Seizures		**m**						
	Unusual sleep patterns		**m**						
Skeletal	Polydactyly	**M**		**m**	**M**		**m**	**M**	
	Short stature			**M**					
	Narrow chest			**M**					
	Brachydactyly			**M**	**M**				
	Micromelia			**M**	**M**			**m**	
	Leg cramps		**M**						
Facial/Oral	Dental abnormalities	**M**							
	Abnormal oral morphology	**M**			**M**		**m**	**m**	
	Dysmorphic facial features				**M**				
Respiratory	Abnormal pattern of respiration						**M**		
	Chronic airway infection		**m**						
	Asthma		**m**						
	Pulmonary hypoplasia							**m**	
	Cystic lung							**m**	

Key features are grouped into 11 body systems. Clinical features marked ‘M’ are major features (present in >50% and/or listed as major diagnostic or characteristic feature in the literature cited). Features marked with ‘m’ are minor features (present in <50% and/or listed as a minor diagnostic feature in the literature cited).

*Feature of *NPHP1-*associated JBTS-plus syndrome (Senior-Loken syndrome).

†Feature of *CEP290*-associated JBTS-plus syndrome (Senior-Loken syndrome, Joubert syndrome with retinal disease, Joubert syndrome with renal disease, COACH syndrome).

‡Feature of *TMEM67*-associated JBTS-plus syndrome (COACH syndrome).

ALMS, Alström syndrome; BBS, Bardet-Biedl syndrome; COACH syndrome, Cerebellar vermis hypoplasia, Oligophrenia, Ataxia, Coloboma and Hepatic fibrosis; EOSRD, early-onset severe retinal dystrophy; JATD, Jeune asphyxiating thoracic dystrophy; JBTS, Joubert syndrome; LCA, Leber congenital amaurosis; m, minor clinical feature; M, major clinical feature; MKS, Meckel-Gruber syndrome; OFD-1, orofaciodigital syndrome 1; SNHL, sensorineural hearing loss.

### Development of a research diagnostic workflow to identify new diagnoses

**Figure 1:**
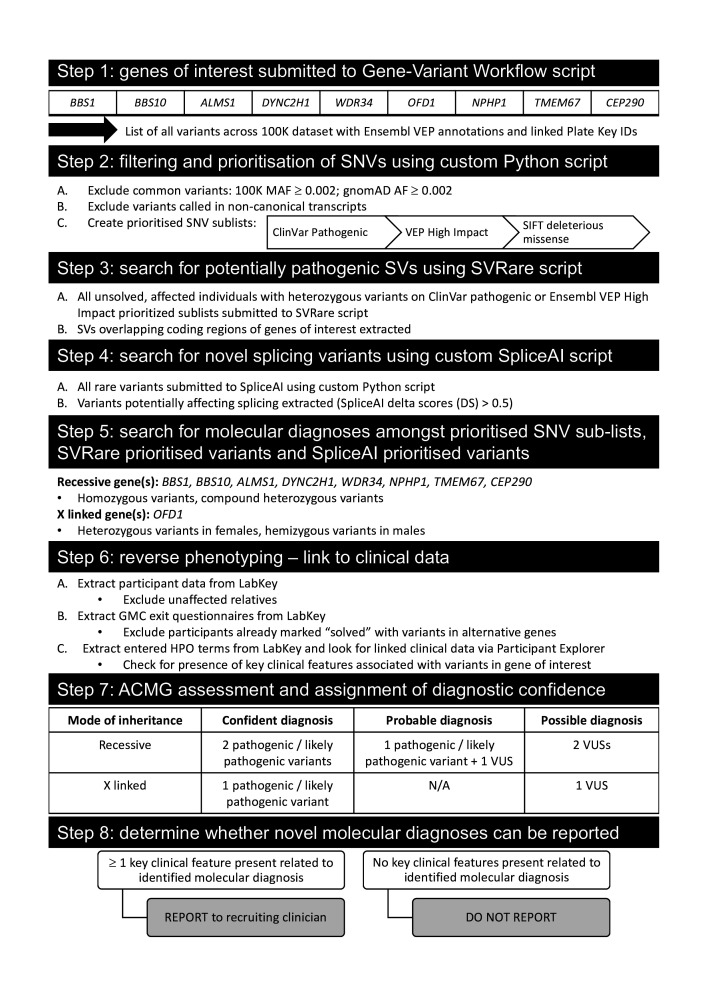
Reverse phenotyping diagnostic research workflow. ACMG, American College of Medical Genetics and Genomics; AF, allele frequency; GMC, Genomics Medical Centres; HPO, Human Phenotyping Ontology; MAF, major allele frequency; N/A, not available; SNV, single nucleotide variant; SV, structural variant; VEP, Variant Effect Predictor; VUS, variant of uncertain significance.

The full diagnostic workflow developed, from extraction through to reporting of variants, is represented in figure 1.

#### Steps 1 and 2: single nucleotide variant filtering and prioritisation

The script ‘Gene-Variant Workflow’ (available from https://research-help.genomicsengland.co.uk/display/GERE/Gene-Variant+Workflow) was used to extract all variants in the nine genes in the 100K dataset from Illumina variant call format (VCF) files, aggregate them together and annotate them using the Ensembl Variant Effect Predictor (VEP).^14^ This includes all intronic and exonic variants within the specified gene region. A custom Python script called filter_gene_variant_workflow.py (available from https://github.com/sunaynabest/filter_100K_gene_variant_workflow) was used to exclude common variants using the following criteria: 100K major allele frequency (MAF) ≥0.002; gnomAD allele frequency (AF) ≥0.002^15^ and variants called in non-canonical transcripts. The allele frequency threshold of 0.002 was calculated using the ImperialCardioGenetics frequency filter calculator (available from https://cardiodb.org/allelefrequencyapp/),^16^ as recommended by the Association for Clinical Genomic Science Best Practice Guidelines.^17^ Parameters were set as follows: biallelic inheritance, prevalence 1 in 500, allelic heterogeneity 0.1, genetic heterogeneity 0.2, penetrance 1, confidence 0.95, reference population size 121 412 (based on the Exome Aggregation Consortium cohort).

Finally, prioritised sublists of SNVs were extracted using filter_gene_variant_workflow.py as follows: (i) ClinVar pathogenic (variants annotated by ClinVar as ‘pathogenic’ or ‘likely_pathogenic’)^18^; (ii) high impact (variants annotated by VEP as ‘high impact’ (stop_gained, stop_lost, start_lost, splice_acceptor_variant, splice_donor_variant, frameshift_variant, transcript_ablation, transcript_amplification)^14^; (iii) SIFT deleterious missenses (missense variants predicted ‘deleterious’ by the in silico prediction tool SIFT).^19^ Additional in silico missense variant predictions were obtained via the Ensembl VEP web interface (available from https://www.ensembl.org/Tools/VEP) from Combined Annotation Dependent Depletion^20^ and PolyPhen-2.^21^

#### Step 3: SVRare script to prioritise potentially pathogenic structural variants

Heterozygous variants in the nine selected genes in either the ‘ClinVar pathogenic’ or ‘high impact’ SNV sublists were then analysed by the SVRare script.^22^ This uses a database of 554 060 structural variants (SVs) called by Manta^23^ and Canvas^24^ aggregated from 71 408 participants in the rare disease arm of 100K. Common SVs (≥10 database calls) were excluded, and the remaining rare SVs that overlapped coding regions of the selected genes were extracted and analysed manually. BAM files for prioritised SVs were inspected in the Integrative Genomics Browser (IGV).^25^ SVs were considered potentially causative if present in >30% of reads. Participants with heterozygous variants identified as ‘deleterious missense’ by SIFT were excluded from further manual analysis by SVRare because of the very high number of such variants and likelihood that they would be classified as VUS. Online supplemental table 4 summarises the numbers of SIFT deleterious missense variant calls in each gene, for example, there are 810 calls in *ALMS1* alone.

#### Step 4: SpliceAI script to prioritise potentially pathogenic splice defects

All rare variants called by the Gene-Variant Workflow script in the nine representative ciliopathy disease genes (100K MAF ≤0.002; gnomAD AF ≤0.002) were run through SpliceAI prediction software with an additional custom Python script (‘find_variants_by_gene_and_SpliceAI_score.py’; available at https://github.com/JLord86/Extract_variants). Variants predicted to affect splicing according to the recommended cut-off (SpliceAI delta scores >0.5) were extracted and analysed manually.^26^ Variants previously annotated by ClinVar as ‘benign’ were excluded.

#### Step 5: search for molecular diagnoses among prioritised variants

All prioritised variant lists were manually analysed for each gene: these comprised ClinVar pathogenic, high impact and SIFT deleterious missense SNV, SVRare and SpliceAI prioritised variant lists. For recessive genes (all except *OFD1*), homozygous or compound heterozygous variants were pursued. Heterozygous variants called in female participants and hemizygous variants called in male participants were pursued for X linked *OFD1*.

#### Step 6: link to clinical data and reverse phenotyping

The Gene-Variant Workflow output files contain) ‘plate key’ identifiers (IDs; unique identifiers used by GEL for DNA sample tracking and logistics) for all participants in whom each variant was called. These unique IDs for participant samples were used to obtain participant data via LabKey, including GMC exit questionnaires reporting outcomes and participant status. Participants were excluded if recruited as unaffected relatives or ‘solved’ or ‘partially solved’ with variants in alternative genes. For remaining participants (all unsolved probands or affected relatives), parental data were analysed where available, to determine variant segregation. HPO terms entered at the time of recruitment were also extracted. Further linked clinical data were obtained using the GEL user interface ‘Participant Explorer’. This links to the source data in LabKey to identify participants with particular clinical phenotypes, determine longitudinal phenotypic and clinical data for any participant and allow comparison between multiple participants. From these, the number of key clinical features related to the identified ciliopathy gene was recorded for each participant, as well as the bodily system(s) involved.

#### Step 7: decision on reporting of novel molecular diagnoses

We reasoned that the presence of at least one major key clinical feature that was compatible with the implicated gene would be sufficient to report any newly identified potential molecular diagnoses to recruiting clinicians. If no major key clinical features were present, we were unable to justify reporting because they could not be considered a potential match for patients’ clinical features, the so-called ‘pertinent findings’.

#### Step 8: ACMG classification and assignment of diagnostic confidence categories for reportable diagnoses

Variant clinical interpretation was reviewed using the American College of Medical Genetics and Genomics (ACMG)/Association for Molecular Pathology guidelines^27^ and each variant of interest among participants with reportable diagnoses was assigned an ACMG pathogenicity score.^17^ Phenotype specificity is a key factor in variant interpretation, so only those deemed potentially pertinent findings, in the presence of at least one major key feature and therefore reportable, underwent variant interpretation and diagnostic confidence scoring. Diagnostic confidence categories were assigned as ‘confident’, ‘probable’ or ‘possible’ based on the assigned ACMG variant classifications (figure 1). A ‘confident’ diagnosis required two pathogenic or likely pathogenic variants in genes with recessive inheritance, or one pathogenic or likely pathogenic variant in *OFD1*. A ‘probable’ diagnosis required one pathogenic/likely pathogenic and one VUS in genes with recessive inheritance; no ‘probable’ classification was possible for *OFD1* variants. A ‘possible’ diagnosis was assigned in the presence of two VUS in recessive genes or one VUS in *OFD1*.

We exported anonymised data for publication through the Airlock system, after review by the GEL Airlock Review Committee. We present only information about the body systems with key features for each participant rather than specific HPO terms, in order to protect participant anonymity.

## Results

### 100K participants previously solved with causative variants in representative ciliopathy disease genes

**Figure 2:**
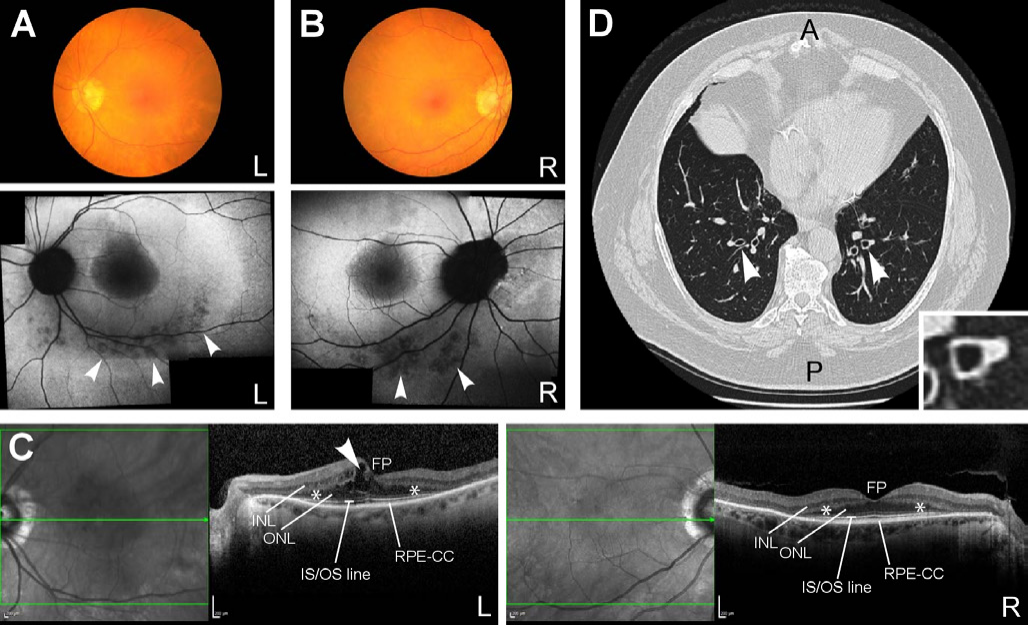
Clinical features of participant #32 consistent with a syndromic ciliopathy. (A) (left eye) and (B) (right eye): upper panels, colour funduscopy of retina; lower panels, fundus autofluorescence images showing perimacular pigment changes (arrowheads) and relatively hypofluorescent central macula. (C) Optical coherence tomography (OCT) for left eye (L; left panel) and right eye (R; right panel), with the plane of OCT shown by green arrows in left-hand regions of each panel, showing loss of ellipsoid zone outside of the central macula with disruption of the outer nuclear later (*) indicative of rod-cone photoreceptor dystrophy. Arrowhead indicates cystoid macular oedema for the left retina. Scale bars=200 µm. (D) CT axial section of chest showing ‘signet ring’ signs (arrowheads; detail shown in inset) typical of bronchiectasis.^46^ A, anterior; FP, foveal pit; INL, inner nuclear layer; IS/OS, inner segment/outer segment; L, left; ONL, outer nuclear layer; P, posterior; R, right; RPE-CC, retinal pigment epithelium-choriocapillaris complex.

Forty-four participants have previously been reported to have ‘solved’ or ‘partially solved’ molecular diagnoses in GMC exit questionnaires with variants in the nine representative ciliopathy disease genes (online supplemental table 3). Seven of these reported cases overlap with participants described in ‘Congenital Malformations caused by Ciliopathies’ cohort analyses.^28^ Interestingly, male participant #32 was reported ‘solved’ with a pathogenic hemizygous *OFD1* frameshift variant in exon 20/23 (NM_003611.3:c.2680_2681del, NP_003602.1:p.(Glu894ArgfsTer6)). Participant #32 was recruited to the ‘rod-cone dystrophy’ category with an apparently milder non-syndromic form of retinal dystrophy that was only identified in late adulthood (online supplemental table 3). Further clinical information from the recruiting clinicians revealed that the participant had a rod-cone dystrophy that lacked bone spicules typical for retinitis pigmentosa but was similar to Bardet-Biedl syndrome (figure 2A, B, C). Participant #32 also had intellectual disability, truncal obesity, evidence of renal failure, short fingers and chronic respiratory disease with mild bronchiectasis (‘signet ring’ signs on CT scan of the chest; figure 2DD). These are clinical features consistent with a syndromic ciliopathy, and we are not aware of any previous reports of males with hemizygous *OFD1* variants having this combination of features.

Molecular details for two reported variants are incomplete, described as a heterozygous ‘large delins’ in *ALMS1* (participant #6) and a ‘whole gene deletion’ of *NPHP1* (participant #33). Data are also incomplete for participant #43, reported solved with a single heterozygous variant, classified as a VUS, in the recessive disease gene *CEP290*.

### New reportable diagnoses identified through the reverse phenotyping research diagnostic workflow

We prioritised a total number of 3666 variants from the SNV, SV and SpliceAI outputs (online supplemental table 4) through our research diagnostic workflow; 30 variants led to potential reportable diagnoses in 18 previously unsolved participants through reverse phenotyping (table 2). However, on further investigation, n=5/18 participants (#45, #47, #48, #50 and #51) had causative variants that were already included in their GMC Exit Questionnaires, but had reporting outcomes annotated as ‘unknown’ or without listing the ciliopathy disease genes of interest. Although these outcomes may be due to inadvertent coding errors, we did not include the data from these participants for further analysis. Our workflow therefore identified a total of n=13/18 participants with new reportable diagnoses.

**Table 2:** Reportable new diagnoses identified via reverse phenotyping research diagnostic workflow

Research ID	Dx confidence	Reported sex	Recruitment category	Gene(s)	Variant zygosity	Consequence	HGVSc	HGVSp	gnomAD AF	100K MAF	SIFT	PolyPhen	CADD	PubMed	ClinVar listing	Segregation	ACMG classification	# of key features	System(s) involved
45	Conf	Ma	Cone dysfunction syndrome	*ALMS1*	Het	FS	NM_015120.4:c.10775del	NP_055935.4:p. Thr3592LysfsTer6	5.23E-05	4.77E-04				11941369, 11941370, 17594715	Path	Pat	Path	1M	M: Oph
					Het	SV	NC_000002.12:g.(73424245_73544334inv ;73424245_73427355dup ;73484777_73544334dup)							Abs	Abs	Mat	Lik_path		
47	Poss	Fe	Bardet-Biedl syndrome	*BBS10*	Hom	Mis	NM_024685.4:c.1790G>A	NP_078961.3:p.Gly597Asp	0	2.54E-05	Delet	Prob_dam		Abs	Abs	Bi-par	VUS	4M	M: Ren, oph, skel, endo/met
48	Conf	Ma	Rod dysfunction syndrome	*NPHP1*	Hom	Mis	NM_001128178.3:c.1027G>A	NP_000263.2:p.Gly343Arg	0.0001155	0.00034312	Delet	Prob_dam	35	10839884	Path	1 par (other unk)	Path	1M, 1m	M: Ren m: Oph
49	Poss	Fe	Cystic kidney disease	*CEP290*	Hom	Intr	NM_025114.4:c.6011+874G>T	–	0	3.81E-05				Abs	Abs	1 par (other unk)	VUS	1M, 1m	M: Ren m: CVS
50	Poss	Fe	Syndromic cleft lip and/or cleft palate	*OFD1*	Het	Mis	NM_003611.3:c.635G>C	NP_003602.1:p.Arg212Pro	0	1.27E-05	Delet	Poss_dam	21.2	Abs	Abs	De novo	VUS	3M	M: Fac/ora (n=2), skel
51	Prob	Ma	Joubert syndrome	*CEP290*	Het	Mis	NM_025114.4:c.104T>G	NP_079390.3:p.Val35Gly	0	1.00E-04	Delet	Prob_dam	33	Abs	Abs	Mat	VUS	4M	M: Oph, neu (n=3)
					Het	SG	NM_025114.4:c.5668G>T	NP_079390.3:p.Gly1890Ter	9.49E-05	2.50E-04				18414213, 26092869, 16682970, 16682973, 17564967	Path	Pat	VUS		
53	Poss	Ma	Cystic kidney disease	*ALMS1*	Het	Mis	NM_015120.4:c.8735A>G	NP_055935.4:p.Gln2912Arg	0	5.00E-05	Delet	Poss_dam	19.03	Abs	Abs	Unk	VUS	2M, 2m	M: Ren, endo/met m: GI, CVS
					Het	Mis	NM_015120.4:c.7412A>G	NP_055935.4:p.Asp2471Gly	0	5.00E-05	Delet	Prob_dam	25.9	Abs	Abs	Unk	VUS		
54	Poss	Fe	Rod-cone dystrophy	*ALMS1*	Het	Mis	NM_015120.4:c.10831A>G	NP_055935.4:p.Arg3611Gly	3.22E-05	5.00E-05	Delet	Poss_dam	23.5	Abs	Abs	Unk	VUS	2M, 1m	M: Oph, ren m: Resp
					Het	Mis	NM_015120.4:c.10377C>G	NP_055935.4:p.Ile3459Met	3.63E-05	5.00E-05	Delet	Poss_dam	23.1	Abs	VUS	Unk	VUS		
55	Conf	Fe	Single autosomal recessive mutation in rare disease	*ALMS1*	Het	FS	NM_015120.4:c.11794del	NP_055935.4:p. Glu3932LysfsTer18	3.99E-06	1.27E-05				Abs	Abs	Unk	Path	4M	M: Oph, endo/met (n=2), CVS
					Het	FS	NM_015120.4:c.1735del	NP_055935.4:p. Arg579GlyfsTer17	1.61E-05	3.18E-05				26104972, 32581362, 17594715, 24462884	Path	Unk	Path		
56	Prob	Ma	Intellectual disability	*ALMS1*	Het	FS	NM_015120.4:c.10775del	NP_055935.4:p. Thr3592LysfsTer6	5.23E-05	0.00047656				11941369	Path	Unk	Path	1M, 2m	M: Sens m: CVS, neu
					Het	Mis	NM_015120.4:c.7510G>T	NP_055935.4:p.Ala2504Ser	8.93E-05	0.00019062	Delet	Prob_dam	25	Abs	VUS	Unk	VUS		
57	Poss	Ma	Congenital hearing impairment	*ALMS1*	Het	Mis	NM_015120.4:c.11429A>G	NP_055935.4:p.Tyr3810Cys	–	1.27E-05	Delet	Prob_dam	27.5	Abs	Abs	Mat	VUS	1M	M: Sens
					Het	Mis	NM_015120.4:c.9148A>G	NP_055935.4:p.Ile3050Val	0.0002007	0.00012708	Delet	Poss_dam	24.2	Abs	VUS	Pat	VUS		
58	Poss	Fe	Syndromic congenital heart disease	*BBS1*	Het	Mis	NM_024649.5:c.734C>T	NP_078925.3:p.Pro245Leu	7.16E-05	6.35E-06	Delet	Ben	23.4	Abs	VUS	Unk	VUS	1M, 1m	M: Neu m: CVS
					Het	Mis	NM_024649.5:c.1313C>G	NP_078925.3:p.Thr438Arg	7.96E-05	4.45E-05	Delet	Prob_dam	25.3	Abs	VUS	Mat	VUS		
62	Conf	Fe	Epilepsy plus other features	*CEP290*	Het	FS	NM_025114.4:c.5434_5435del	NP_079390.3:p. Glu1812LysfsTer5	1.14E-05	4.45E-05				Abs	Path	Unk	Path	2M	M: Oph, Neu
					Het	Intr	NM_025114.4:c. 2991+1655A>G	–	0	0.00040031				20301475, 17964524, 20301500, 16909394, 17564967, 17345604	Path	Unk	Path		
63	Conf	Fe	Cystic kidney disease	*CEP290*	Het	SL	NM_025114.4:c.2T>A	NP_079390.3:p.Met1?	4.07E-06	2.54E-05				Abs	path/lik _path	Pat	Path	2M	M: Ren, oph
					Het	SG	NM_025114.4:c.4966G>T	NP_079390.3:p.Glu1656Ter	3.60E-05	1.59E-04				23559409, 25525159, 16909394, 20079931	path/lik _path	Mat	Path		
66	Poss	Ma	Leber congenital amaurosis or early onset severe retinal dystrophy	*CEP290*	Hom	Mis	NM_025114.4:c.182T>C	NP_079390.3:p.Met61Thr	–	2.54E-05	Delet	Poss_dam	25.6	Abs	Abs	Bi-par	VUS	3M	M: Oph, neu (n=2)
67	Poss	Fe	Ultra-rare undescribed monogenic disorders	*CEP290*	Hom	Mis	NM_025114.4:c.5284C>T	NP_079390.3:p.Arg1762Cys	3.78E-05	9.53E-05	Delet	Poss_dam	29.6	25741868	VUS	Unk	VUS	2M, 1m	M: Oph, neu m: GI
70	Conf	Fe	Proteinuric renal disease	*DYNC2H1*	Het	Syn	NM_001377.3:c.11049G>A	NP_001368.2:p.Pro3683=	1.21E-05	0.00014996				Abs	Lik_path	Pat	Lik_path	2M	M: Ren, skel
					Het	SV	NC_000011.9:g. 103445518_10350188del							Abs	Abs	Mat	Lik_path		
75	Poss	Ma	Distal myopathies	*TMEM67*	Het	Mis	NM_153704.6:c.2035G>C	NP_714915.3:p.Glu679Gln	0	5.00E-05	Delet	Prob_dam	26.5	Abs	Abs	Unk	VUS	1M	M: Ren
					Het	Mis	NM_153704.6:c.755T>C	NP_714915.3:p.Met252Thr	8.36E-05	2.00E-04	Delet	Ben	23.7	26092869, 19508969, 21866095	Path	Unk	Path		

Abs, absent ; ACMG, American College of Medical Genetics and Genomics; AF, allele frequency; Bi-par, bi-parental; CADD, Combined Annotation Dependent Depletion; Conf, confident; Dx, diagnostic; Endo/met, endocrine/metabolic; Fac/ora, facial/oral; Fe, female; FS, frameshift; Hemi, hemizygous; Het, heterozygous; HGVSc, Human Genome Variation Society coding; HGVSp, Human Genome Variation Society protein; Hom, homozygous; Intr, intronic; 100K, 100 000 Genomes Project; Lik_path, likely pathogenic; M, major clinical feature; m, minor clinical feature; Ma, male; MAF, maximum allele frequency; Mat, maternal; Mis, missense; Neu, neurological; Oph, ophthalmic; 1 par (other unk), 1 parent, other unknown; Pat, Paternal; Path, pathogenic; Poss, possible; Prob, probable; Spl Reg, splice region; Ren, renal; Resp, respiratory; Sens, sensory; SG, stop gain; Skel, skeletal; SL, start loss; Spl A, splice acceptor; SV, structural variant; Syn, synonymous; Unk, unknown; VUS, variant of uncertain significance.

### Identification of reportable SVs

**Figure 3:**
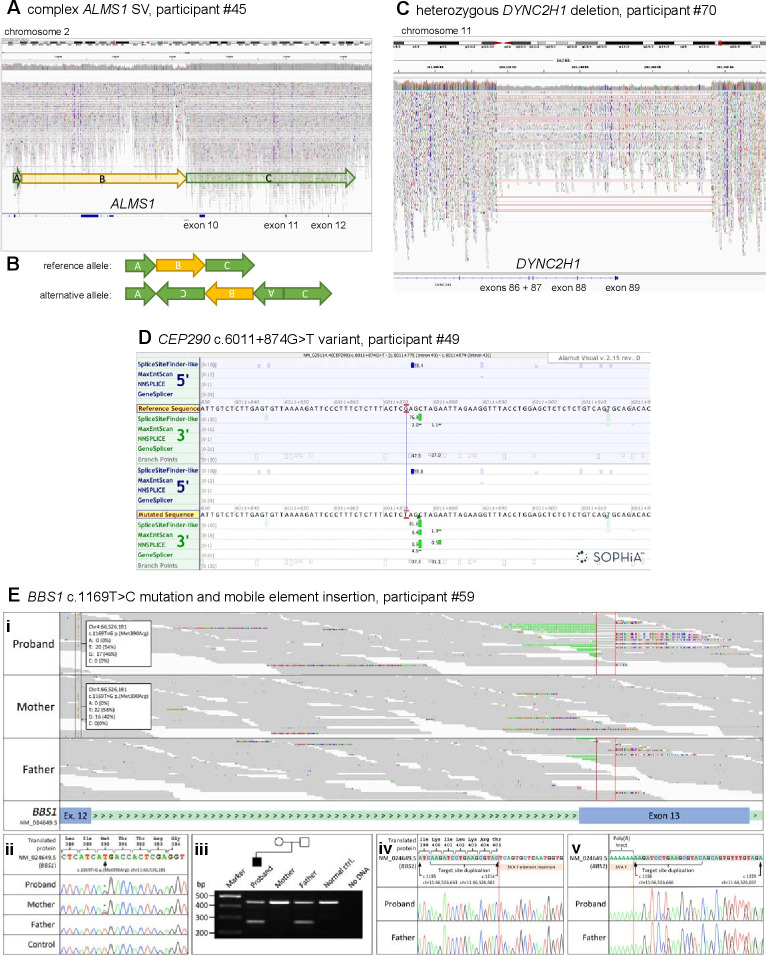
Likely pathogenic structural variants and other variants in selected ciliopathy genes identified through the reverse phenotyping research diagnostic workflow. (A) IGV plot of *ALMS1* (NM_015120.4) in participant #45. We observed a monoallelic complex SV in the *ALSM1* gene spanning from chr2:g.73424245 to chr2:g.73544334 (GRCh38). (B) Diagrammatic representation of complex *ALMS1* SV in participant #45. After inspection of the IGV plots, we surmised that the alternative allele is a paired-duplication inversion, with block A at chr2:g.73424245_73427355, covering exons 4 and 5 (NM_015120.4), block B at chr2:g.73427355_73484777, covering exons 6–9 and block C at chr2:g.73484777_ 73 544 334. Note that the boundary between block B and C is an estimate as it is within a region with relatively low alignment quality. (C) IGV plot of heterozygous 56 kb deletion identified in *DYNC2H1* (NM_001377.3) in participant #70. The terminal four exons (86–89) have been deleted. (D) Alamut screenshot for *CEP290* c.6011+874G>T variant in participant #49. Top tracks are donor/acceptor splice site predictions for the reference sequence and the bottom tracks are donor/acceptor predictions for the mutated sequence. Green highlighting identifies increasing scores for a potential splice acceptor site in the non-reference mutated sequence track. (E) Analysis of the *BBS1* locus for Congenital Malformations caused by Ciliopathies (CMC) cohort participant #59 following trio whole genome sequencing. (i) The maternally inherited pathogenic variant, NM_024649.5:c.1169T>G, NP_078925.3:p.(Met390Arg) (highlighted by the black frames) is in trans with a paternally inherited mobile element insertion for which the target site duplication sequence is highlighted (red frames). Soft-clipped junction spanning reads, showing inserted nucleotides and the terminal poly(A) tract, are visible. (ii) Sanger sequencing confirmation of the maternally inherited c.1169T>C mutation. Exon 12 coding sequence is highlighted in peach. (iii) Duplex screening assay^32^ confirming that the mobile element insertion was present in the proband and his father (270 bp band). Upstream (iv) and downstream (v) junction fragments confirm that the target site duplication sequence is as previously reported.^32^ Exon 13 coding sequence is highlighted in grey. Genomic coordinates are according to Human Genome build Hg38. Variant nomenclature is according to transcript NM_024649.5. IGV, Integrative Genomics Browser; SV, structural variant.

Two participants have been identified with new potentially causative SVs through the SVRare script (figure 3). Participant #45 had a maternally inherited, 116 969 bp chr2 inversion and a 63 550 bp gain (identified using Manta and Canvas, respectively), both including coding regions of *ALMS1*. After a careful inspection of the IGV plot, we also observed a monoallelic, complex SV in the *ALSM1* gene spanning from chr2: g.73424245 to chr2: g.73544334 (GRCh38). We interpreted this as a paired-duplication inversion (figure 3A–B). Ideally, this would be confirmed experimentally; we have contacted the recruiting clinician about performing these studies but no response has been received. Participant #45 also has a paternally inherited, known pathogenic *ALMS1* frameshift variant (NM_015120.4:c.10775del, NP_055935.4:p.Thr3592LysfsTer6). Therefore, segregation analysis is consistent with autosomal recessive inheritance as expected. Participant #45 was recruited to the cone dysfunction category and has one *ALMS1* key feature involving the ophthalmic system that allowed this research finding to be reported to the recruiting clinician.

Participant #70 had a maternally inherited, 56 371 bp chromosome 11 deletion (identified by Canvas), including the terminal four exons of *DYNC2H1* (figure 3C). This individual also has a ClinVar ‘likely pathogenic’ paternally inherited *DYNC2H1* synonymous variant (NM_001377.3: c.11049G>A, NP_001368.2: p.Pro3683=). This variant is predicted to cause a splice acceptor loss by SpliceAI (DS_AL 0.51). No clinical detail is provided with the ClinVar entry (from the Rare Disease Group, Karolinska Institutet), but the ‘likely pathogenic’ listing in association with Jeune syndrome provides some confidence in this assessment of pathogenicity. Participant #70, recruited to the proteinuric renal disease category, has two Jeune syndrome key features from the renal and skeletal systems, allowing this research finding to be reported to the recruiting clinician. Furthermore, the participant’s affected sibling, also recruited to 100K with three Jeune syndrome clinical key features from the renal and skeletal systems, was found to have the same two variants, strengthening the confidence in the diagnosis.

### Identification of reportable non-canonical splice defects

One new homozygous *CEP290* intronic variant has been identified by using our SpliceAI script, predicted to cause a splice acceptor gain (SpliceAI DS_AG 0.64) (NM_025114.4:c.6011+874G>T) and gain of a potential splice acceptor site (Alamut screenshot; figure 3D). This variant was identified in participant #49, recruited to the cystic kidney disease category. The proband’s father is heterozygous for the variant, but there is no maternal sample available in 100K. The recruiting clinician has been contacted and relevant tissues (blood, urinary renal epithelial cells) requested to perform functional splicing assays, but no response has been received. Therefore, the variant has been called a VUS, allowing classification of only a ‘possible’ diagnosis to be made.

### Novel unreportable diagnoses identified through research workflow

Eleven participants have unreportable, novel diagnoses in the nine ciliopathy disease genes (table 3). These participants have no major key clinical features among their entered HPO terms, or identifiable among the additional clinical data available on Participant Explorer, that can justify reporting to recruiting clinicians as potentially pertinent clinical findings. Four of these 11 have novel missense variants, which can only be classified as VUS. The other seven (#60, #61, #64, #65, #71, #72, #73) have at least one more definitively damaging variant, including high impact frameshifts, stop gains, splice acceptors and ClinVar pathogenic missenses.

**Table 3:** Novel, unreportable diagnoses identified via reverse phenotyping research diagnostic workflow

Research ID	Recruitment category	Gene	Variant zygosity	Consequence	HGVSc	HGVSp	gnomAD AF	100K MAF	SIFT	PolyPhen	CADD	PubMed	ClinVar listing	Segregation	# of key features
52	Intellectual disability	*ALMS1*	Het	Mis	NM_015120.4:c.7738A>T	NP_055935.4:p.Ile2580Phe	4.01E-06	0.00009997	Delet	Ben	22.5	Abs	Abs	Mat	0M, 2m
			Het	Mis	NM_015120.4:c.346C>T	NP_055935.4:p.His116Tyr	2.41E-05	0.00019994	Delet	Ben	16.05	Abs	Abs	Pat	
59	Hereditary spastic paraplegia	*BBS1*	Het	Mis	NM_024649.5:c.235G>A	NP_078925.3:p.Glu79Lys	0.000756	0.00120728	Delet	Poss_dam	23.8		VUS	Unk	0M, 0m
			Het	Mis	NM_024649.5:c.1714G>T	NP_078925.3:p.Gly572Cys	–	1.27E-05	Delet	Prob_dam	32		Abs	Unk	
60	Primary immunodeficiency	*CEP290*	Het	FS	NM_025114.4:c.6154_6161del	NP_079390.3:p.Asp2052LeufsTer17	0	1.27E-05				Abs	Abs	Unk	0M, 0m
			Het	FS	NM_025114.4:c.7412_7415del	NP_079390.3:p.Glu2471ValfsTer13	0	1.91E-05				Abs	Abs	Unk	
61	Primary lymphoedema	*CEP290*	Het	SG	NM_025114.4:c.7048C>T	NP_079390.3:p.Gln2350Ter	1.90E-05	1.91E-05				Abs	lik_path	Unk	0M, 0m
			Het	Mis	NM_025114.4:c.4063C>T	NP_079390.3:p.Arg1355Cys	4.97E-05	9.53E-05	Delet	Prob_dam	32	Abs	VUS	Unk	
64	Limb-girdle muscular dystrophy	*CEP290*	Hom	Mis	NM_025114.4:c.4805C>T	NP_079390.3:p.Thr1602Met	0.000226	0.00027958	Delet	Poss_dam	27.7	Abs	lik_path, VUS	Unk	0M, 0m
65	Undiagnosed monogenic disorders	*CEP290*	Het	Mis	NM_025114.4:c.5909C>A	NP_079390.3:p.Thr1970Asn	0	1.27E-05	Delet	Prob_dam	25.6	Abs	Abs	Unk	0M, 0m
			Het	SG, FS	NM_025114.4:c.7283_7286dup	NP_079390.3:p.Tyr2429Ter	2.11E-05	2.54E-05				Abs	Abs	Unk	
68	Early onset dementia	*CEP290*	Het	Mis	NM_025114.4:c.31A>G	NP_079390.3:p.Met11Val	7.72E-05	6.35E-06	Delet	Ben	23.2	–	VUS	Unk	0M, 0m
			Het	Mis	NM_025114.4:c.2447G>A	NP_079390.3:p.Arg816His	3.13E-05	6.35E-05	Delet	Prob_dam	26.4	–	VUS	Unk	
69	Epilepsy plus other features	*CEP290*	Het	Mis	NM_025114.4:c.2446C>T	NP_079390.3:p.Arg816Cys	5.00E-05	2.54E-05	Delet	Prob_dam	32	25 741 868	VUS	Unk	0M, 0m
			Het	Mis	NM_025114.4:c.4741C>T	NP_079390.3:p.Leu1581Phe	1.32E-05	1.27E-05	Delet	Poss_dam	25.1	25 741 868	VUS	Unk	
71	Hereditary ataxia	*DYNC2H1*	Het	Mis	NM_001377.3:c.10142C>T	NP_001368.2:p.Pro3381Leu	3.62E-05	1.00E-04	Delet	Prob_dam	31	Abs	lik_path, path	Unk	0M, 0m
			Het	Mis	NM_001377.3:c.3419G>T	NP_001368.2:p.Gly1140Val	0.0003938	0.00044987	Delet	Ben	23.2	Abs	VUS	Unk	
72	Early onset dementia	*OFD1* (♀)	Het	Spl_A	NM_003611.3:c.936–1G>A	–	0	6.35E-06				Abs	Abs	Unk	0M, 0m
73	Early onset dystonia	*OFD1* (♀)	Het	FS	NM_003611.3:c.1911del	NP_003602.1:p.Glu637AspfsTer29	0	6.35E-06				Abs	Abs	Unk	0M, 0m

Abs, absent; ACMG, American College of Medical Genetics and Genomics; AF, allele frequency; CADD, Combined Annotation Dependent Depletion; F, female; FS, frameshift; Hemi, hemizygous; Het, heterozygous; HGVSc, Human Genome Variation Society coding; HGVSp, Human Genome Variation Society protein; Hom, homozygous; Intr, intronic; 100K, 100 000 Genomes Project; Lik_path, likely pathogenic; M, male; M, major clinical feature; m, minor clinical feature; MAF, maximum allele frequency; Mat, maternal; Mis, missense; Pat, paternal; Path, pathogenic; Spl Reg, splice region; SG, stop gain; SL, start loss; Spl A, splice acceptor; SV, structural variant; Syn, synonymous; Unk, unknown; VUS, variant of uncertain significance.

## Discussion

### Reportable diagnoses

We have used a reverse phenotyping strategy to identify 62 reportable molecular diagnoses with variants in 9 prioritised, multisystemic ciliopathy genes (*BBS1, BBS10, ALMS1, OFD1, DYNC2H1, WDR34, NPHP1, TMEM67, CEP290*). The nine genes chosen were representative exemplars that, from the literature review, span the extensive phenotypic range of ciliopathies. The addition of other ciliopathy genes (such as *CPLANE1* for JBTS) would, of course, further increase diagnostic yield. Forty-four have been previously reported by 100K in GMC Exit Questionnaires, 5 were previously unreported and 13 represent new diagnoses that are compatible with the entered clinical features for unsolved participants (table 2). Based on ACMG classifications of underlying variants, 6 are classified as confident diagnoses, 2 as probable diagnoses and 10 as only possible diagnoses. In summary, 14 molecular diagnoses are in *ALMS1*, 13 in *BBS1*, 2 in *BBS10*, 16 in *CEP290*, 3 in *DYNC2H1*, 7 in *OFD1*, 4 in *NPHP1* and 3 in *TMEM67*. No molecular diagnoses have been made in *WDR34*. These ciliopathy findings fit with what has previously been reported for reverse phenotyping studies; namely, that this approach proves particularly useful in conditions with high genetic heterogeneity and/or complex phenotypes.^4–6^

We have reported VUS results to recruiting clinicians in this project by using RID forms submitted through the secure GEL Airlock. The ACMG advises that VUS results cannot be used in clinical decision-making.^27^ This applies to the index patient, and to cascade testing of other family members and to prenatal testing. If reported to patients, VUS can cause significant anxiety and make decision-making challenging.^22 29^ We do not anticipate that VUS results identified through this study will be immediately reported back to patients by recruiting clinicians, but there is a high probability that at least some are the correct molecular diagnosis. Therefore, we believe it is important to report them from the research setting for current and future consideration, especially with the emergence of improved functional variant interpretation tools. The problem lies is the lack of available lines of evidence to perform definitive variant classification, especially for missense and splice variants. The ACMG advises that ‘efforts to resolve the classification of the variant as pathogenic or benign should be undertaken’ when VUS are identified.^27^ Currently, functional work to provide additional ‘strong’ evidence is largely limited to the research setting, done on a case-by-case basis where resources are available and interested researchers are involved. Improved variant sharing will also facilitate better variant classification because the recurrent identification of potential disease alleles among individuals with convincing shared phenotypes adds weight to the assessment of variant pathogenicity.

Alström syndrome is one of the rarer ciliopathies, with an estimated prevalence of 1:100 000 to 1:1 000 000 and around 950 affected individuals reported worldwide.^30^ Biallelic *ALMS1* variants have recently also been published as rare causes of non-syndromic retinal dystrophy and cardiomyopathy (online supplemental table 1). The identification of 14 patients with biallelic, predicted pathogenic *ALMS1* variants is therefore higher than anticipated and may reflect under-recognition of this disease gene in the clinical setting. We expected to find a higher rate of *TMEM67* diagnoses than the three identified, given that *TMEM67* is the leading cause of JBTS and MKS, and is also associated with NPHP and COACH syndrome (online supplemental table 1). Potentially, given the greater disease burden and therefore familiarity with *TMEM67,* more straightforward *TMEM67* diagnoses may have been identified by mainstream testing, preventing the need for those participants to be enrolled into 100K. However, this explanation may not be true for other genes because all 12 of the GEL reported *BBS1* cases had at least one copy of the founder missense variant NM_024649.5:c.1169T>G, NP_078925.3:p.(Met390Arg). This is known to be the most frequent cause of BBS,^31^ and it would be expected to be identified by routine testing.

To illustrate the challenge of diagnosing biallelic *BBS1* variants, even when one copy is the common founder missense variant NM_024649.5:c.1169T>G, NP_078925.3:p.(Met390Arg), we further reviewed the ‘Congenital Malformations caused by Ciliopathies’ (CMC) cohort, as described previously,^13^ for potential compound heterozygous *BBS1* variants. Through manual inspection of aligned sequence reads in IGV, we identified a soft-clipped read signature in exon 13 in CMC cohort participant #59 (figure 3E) that was consistent with a recently described mobile SVA F family element insertion of size 2.4 kb.^32^ Analysis of parental alignments supported the variant being in trans with the maternally inherited c.1169T>C missense mutation (figure 3E). A duplex PCR screening assay (online supplemental data 1) and sequencing confirmed the presence of the mobile element insertion in the proband and their father (figure 3E). This case further demonstrates that reanalysis of 100K data improves diagnostic yield, and allows refinement (online supplemental data 1) of an existing diagnostic screening strategy^32^ that allows interpretation of unusual alignment profiles in short-read sequencing datasets.

### Sources of additional diagnoses from the reverse phenotyping research diagnostic workflow

The Genomics England Rare Disease Tiering Process includes an automated variant triaging algorithm to classify variants on virtual PanelApp panel(s) selected according to entered HPO terms into a series of ‘Tiered’ categories, which have been described previously.^2 33^ Tiered variants are primarily limited to those variants affecting coding sequences, and splice donor or acceptor sites. The standard 100K pipeline requires diagnostic labs to analyse variants that are triaged into tier 1 or 2. Tier three variants (rare coding SNVs in genes not included in the selected panel or panels) and untiered variants are not routinely analysed in the diagnostic setting. The selection of incorrect panels that prevents appropriate tiering of causative variants, and the fact that certain types of variant are not routinely tiered, will therefore both contribute to missed diagnoses. Furthermore, inaccurate or incomplete HPO term entries at the time of recruitment will lead to inappropriate virtual gene panel selections that will not allow the analysis of the correct causative disease gene. These problems of missed diagnoses for both the present reverse phenotyping study and our previous analysis of the ‘CMC’ cohort,^13^ suggests that a change in protocol should be considered. This would permit further gene panel selection in the absence of good phenotyping data, or when the answer is not found from the first panel(s) applied.

SVs and single heterozygous SNVs in recessive disease genes are not routinely tiered, even when the genes are on the panel(s) applied. Filtering of all variants in our selected genes independent of the GEL tiering system, followed by independent annotation and analysis, has allowed us to identify SNVs most likely to be pathogenic, even when they are a single hit in a recessive disease gene. If the second variant in the same gene is difficult to find, for example, if it is an SV or intronic variant, then their identification in our pipeline could improve diagnostic yield. In particular, the introduction of the SVRare script,^22^ permitting exclusion of SV calls from analysis if they appear in >10 100K participants, has facilitated diagnosis of two previously unsolved participants (#45 and #70) with untiered, likely pathogenic SVs. SVRare provides a fast and systematic approach to SV analysis, which will be invaluable for future genomic studies. All 100K participants have SV.vcf files available in the Research Environment, called using the Manta and Canvas pipelines.^23 24^ To date, strategies to filter the huge number of SVs from these outputs, most of which are common and benign, have been limited. Alongside manual IGV inspection, the SVRare pipeline also allowed more accurate definition of the complex *ALMS1* SV found in participant #45, since it was called as both a rare inversion (Manta) and duplication (Canvas).

A further source of untiered, potentially pathogenic variants is our custom SpliceAI script. Currently, novel intronic variants are not routinely tiered. SpliceAI has provided one possible new diagnosis in participant #49, with the identification of a rare, homozygous intronic variant predicted to cause a *CEP290* splice acceptor gain (NM_025114.4:c.6011+874G>T, SpliceAI DS_AG 0.64; figure 3D).

These sources of potentially missed causative variants shows the value of research collaborations to make the most of available genomic data. In particular, comprehensive SV and intronic variant analysis facilitates diagnoses not easily achievable through WES and gene-panel testing, but the standard 100K diagnostic pipelines do not yet take full advantage of these analyses.

### The challenge of poor phenotyping data that prevents accurate variant interpretation

The quality of phenotyping has proven highly significant in determining the accuracy of variant interpretation in this study. At the time of recruitment to 100K, the HPO term entry for participants was frequently sparse, comprising one or two terms only, often from just one organ system. The Participant Explorer user interface can provide additional clinical data from longitudinal patient records, which summarise medical history, and timelines for inpatient and outpatient observations, treatments and procedures. However, these data are of variable quality, and clinical features are not collated in a form amenable for genotype-phenotype correlation analyses. Given the frequently sparse clinical data available, we decided to report identified molecular diagnoses among participants with at least one major key clinical feature. This was to maximise the number of potential new diagnoses. With the limited data and systems available, we must pass responsibility on to the recruiting clinicians to refine any phenotypic fit in light of any additional clinical data to which they have access.

Effective communication with recruiting clinicians, providing additional clinical information not entered at the time of recruitment to 100K, has proven invaluable for accurate variant interpretation. However, of the 20 researcher-identified diagnosis forms and clinical collaboration request forms submitted via the GEL Airlock in the last 3 months, we have only received responses from four recruiting clinicians. Participant #62, recruited under the ‘epilepsy plus other features’ category with an ‘unsolved’ status on their GMC exit questionnaire, illustrates the value of effective researcher-clinician collaboration. We identified a ClinVar pathogenic *CEP290* frameshift variant (NM_025114.4:c.5434_5435del, NP_079390.3:p.Glu1812LysfsTer5) and a deep intronic *CEP290* variant known to cause a strong splice-donor site and insertion of a cryptic exon (NM_025114.4:c.2991+1655A>G).^34^ Participant #62 had one *CEP290-*related key clinical feature from the ophthalmic system category (keratoconus), permitting us to report the finding. The recruiting clinician confirmed the presence of key ophthalmological features not entered during recruitment to 100K, comprising a formal diagnosis of Leber Congenital Amaurosis (bilateral keratoconus and cataracts, no detectable ERG responses to light) that was not previously specified. This strengthened confidence that the molecular diagnosis is correct and that this participant is highly likely to have a *CEP290*-related syndromic ciliopathy. It is unclear if the neurological features reported for participant #62 (diffuse cerebellar atrophy confirmed by MRI, but no evidence of structural brain abnormalities or intellectual disability), in addition to epilepsy, are associated with syndromic ciliopathy or comprise a separate phenotype. Nevertheless, reporting the molecular diagnosis is especially important in this instance, because the *CEP290* c.2991+1655A>G variant is a target for the development of antisense oligonucleotides that may offer a personalised therapy for patients.^35 36^

### Reverse phenotyping facilitates expansion of ciliopathy disease-gene associations

As was previously demonstrated for a family with an *INPP5E*-related ciliopathy,^6^ this study widens the phenotypic spectrum of known ciliopathy disease-gene associations through reverse phenotyping. For example, male participant #32 was reported ‘solved’ with a pathogenic hemizygous *OFD1* frameshift variant in exon 20/23 (NM_003611.3:c.2680_2681del, NP_003602.1:p.Glu894ArgfsTer6). Although participant #32 was recruited to the ‘rod-cone dystrophy’ category with apparently non-syndromic retinal dystrophy, reverse phenotyping revealed that he had clinical features that were consistent with a syndromic ciliopathy. Truncating variants in the C-terminal end of *OFD1* (exons 20–21) have recently been associated with the motile ciliopathy primary ciliary dyskinesia (PCD) without the characteristic skeletal, neurological or renal features of other *OFD1*-related disorders.^32 37^ The OFD1 protein is a component of ciliary basal bodies and centrioles, and has been shown to be essential for both primary and motile ciliogenesis.^38^ Therefore, it is entirely plausible that pathogenic *OFD1* variants could cause features compatible with both motile and primary ciliopathies, therefore accounting for participant #32’s full constellation of features (retinal dystrophy, renal failure and intellectual disability in keeping with primary ciliopathies and PCD-like respiratory disease with motile ciliopathies). Further reports of patients with both motile and primary ciliopathy features that carry pathogenic *OFD1* variants would strengthen this potential broadening of associated phenotypes. It is possible that the exon 20 frameshift variant identified in participant #32 could just explain part of his phenotype, for example, his PCD-like respiratory disease, in keeping with the published literature.^32 37^ Conversely, retinal dystrophy may be an additional feature, as has been reported in association with X linked recessive JBTS caused by pathogenic *OFD1* variants in affected males.^39^ We therefore suggest that individuals with a suspected *OFD1*-associated ciliopathy undergo a formal ophthalmological assessment to strengthen the diagnosis.

### Unreportable diagnoses

As well as the 18 reportable molecular diagnoses, we also identified 11 unreportable molecular diagnoses for the 9 ciliopathy disease genes (table 3). Parental sequence is not available for any of the participants with unreportable diagnoses apart from one (#52). Lack of segregation analyses hamper accurate variant interpretation. Nevertheless, it is highly likely that some of these molecular diagnoses are correct and clinically actionable, with implications for the proband and for their relatives. The inability to report these findings is likely to be driven by inaccurate HPO term entry, which is a great loss to the participants. A review of reporting guidelines, given this important observation, may prove beneficial. For example, a system could be devised that marks potential pathogenic variants of interest that then requests further clinical information, but these remain unreportable until further, actionable data are available.

### Conclusion

This study reveals the power of reverse phenotyping approaches to improve diagnosis rates for rare disease participants entered into large-scale genomic studies such as the 100K. Through the application of additional novel screening methodologies such as the SVRare suite, and with domain-specific knowledge, we have confirmed existing ciliopathy diagnoses and identified additional ones in a series of 100K participants who were not originally recruited as having a primary ciliopathy. Our findings suggest that diagnoses may be missed when screening of limited gene panels is directed by incorrect or incomplete HPO term entry, and that inaccurate phenotyping may prevent participants from accessing clinically valuable findings. We have discussed the challenges of 100K analyses more extensively in our recent commentary article and suggest potential improvements for future use of 100K data.^33^ Clearly, open dialogue between researchers, clinicians and clinical scientists is essential to fully exploit the available data for patient benefit in the postgenomic era.

## Data Availability

Data are available in a public, open access repository. Data are available on reasonable request.

## Data availability statement

Full data are available in the Genomic England Secure Research Environment. All datasets are available in the re_gecip shared folder of the GEL research environment for approved researchers. Access to our folder containing variant data (re_gecip/shared_allGeCIPs/GW_SB) can be requested from the GEL Helpdesk.
