# Supplementary material for: Uncovering the burden of hidden ciliopathies in the 100 000 Genomes Project: a reverse phenotyping approach
Source: J Med Genet. 2022 Jun 28;59(12):1151–64. doi: 10.1136/jmedgenet-2022-108476 (PMC9691823; doi:10.1136/jmedgenet-2022-108476)
Supplement: Supplementary data [file jmedgenet-2022-108476supp001.pdf]

BMJ Publishing Group Limited (BMJ) disclaims all liability and responsibility arising from any reliance placed on this supplemental material which has been supplied by the author(s)

Supplementary Table 1: Selection of leading multi-systemic ciliopathy disease genes from the medical literature

**Ciliopathy syndrome**	**Leading genetic cause(s)**	**Mode of inheritance**	**Further ciliopathies associated with gene**	**Reference(s)**
Bardet-Biedl syndrome (BBS)	*BBS1* (23.4% of all BBS)	Recessive	N/A	(1-3)
	BBS10 (14.5% of all BBS)	Recessive	N/A	
Alström Syndrome (ALMS)	*ALMS1* (only causative gene)	Recessive	-Non-syndromic retinal dystrophy<br>-Non-syndromic cardiomyopathy	(4-8)
Joubert syndrome (JBTS) and Meckel Gruber syndrome (MKS)	*TMEM67* (6-26% of all JBTS; 16% of all MKS)	Recessive	-NPHP with hepatic fibrosis<br>-COACH syndrome (cerebellar vermis hypo/aplasia, oligophrenia, ataxia, ocular coloboma, and hepatic fibrosis)	(9-17)
	CEP290 (6-22% of all JBTS, 2nd most common cause of MKS)	Recessive	-Leber Congenital Amaurosis (LCA) / Early-Onset Severe Retinal Dystrophy (EOSRD) (15-20% of LCA / EOSRD cases)<br>-NPHP<br>-BBS<br>-Senior-Løken syndrome<br>-COACH syndrome	(14, 18-24)
Jeune Asphyxiating Thoracic Dystrophy (JATD)	*DYNC2H1* (~50% of all JATD)	Recessive	N/A	(25-28)
	WDR34 (~10% of all JATD)	Recessive		
Nephronophthisis (NPHP)	*NPHP1* (20-25% of all NPHP)	Recessive	JBTS	(29-31)
Oral-facial-digital syndrome (OFD) Type 1	*OFD1* (only genetic cause)	X-linked dominant	JBTS (X-linked recessive)	(9, 32)

BMJ Publishing Group Limited (BMJ) disclaims all liability and responsibility arising from any reliance placed on this supplemental material which has been supplied by the author(s)

Supplementary Table 2: HPO terms linked to clinical key terms for ciliopathy syndromes

Key term	HPO ID	HPO descriptor	Linked HPO terms included in analysis
Retinal dystrophy	HP:0000556	Breakdown of light-sensitive cells in back of eye	• Cone/cone-rod dystrophy + sub-terms<br>• Rod-cone dystrophy + sub-terms<br>• Pattern dystrophy of the retina + sub-terms
Abnormality of eye movement	HP:0000496	An abnormality in voluntary or involuntary eye movements or their control	• Oculomotor apraxia (JBTS)<br>• Nystagmus (LCA)<br>• Roving eye movements (LCA)
Abnormal renal morphology / renal insufficiency	HP:0012210	Any structural anomaly of the kidney	• Abnormal localisation of kidney + sub-terms<br>• Abnormal renal cortex morphology + sub-terms<br>• Abnormal renal echogenicity + sub-terms<br>• Abnormal renal medulla morphology + sub-terms<br>• Abnormal renal pelvis morphology + sub-terms<br>• Renal cyst + sub-terms<br>• Renal dysplasia + sub-terms<br>• Renal fibrosis + sub-terms<br>• Renal hypoplasia/aplasia + sub-terms
	HP:0000083	A reduction in the level of performance of the kidneys in areas of function comprising the concentration of urine, removal of wastes, the maintenance of electrolyte balance, homeostasis of blood pressure, and calcium metabolism	• Chronic kidney disease + sub-terms
Abnormality of the liver	HP:0001392	An abnormality of the liver	• Abnormal liver morphology + sub-terms<br>• Abnormal liver physiology + sub-terms<br>• Abnormality of the biliary system + sub-terms
Abnormality of the genitourinary system	HP:0000119	The presence of any abnormality of the genitourinary system	• Abnormality of the genital system + sub-terms<br>• Abnormality of the urinary system + sub-terms
Cardiomyopathy	HP:0001638	A myocardial disorder in which the heart muscle is structurally and functionally abnormal, in the absence of coronary artery disease, hypertension, valvular disease and congenital heart disease sufficient to cause the observed myocardial abnormality.	• All sub-terms
Sensorineural hearing impairment	HP:0000407	A type of hearing impairment in one or both ears related to an abnormal functionality of the cochlear nerve.	• All sub-terms

Abnormality of the sense of smell	HP:0004408	An anomaly in the ability to perceive and distinguish scents (odors).	• All sub-terms
Abnormal pattern of respiration	HP:0002793	An anomaly of the rhythm or depth of breathing	• Apnoea + sub-terms<br>• Tachypnoea + sub-terms
Hypogonadotrophic hypogonadism	HP:000044	Hypogonadotropic hypogonadism is characterized by reduced function of the gonads (testes in males or ovaries in females) and results from the absence of the gonadal stimulating pituitary hormones: follicle stimulating hormone (FSH) and luteinizing hormone (LH).	• All sub-terms
Glucose intolerance	HP:0001952	Glucose intolerance (GI) can be defined as dysglycemia that comprises both prediabetes and diabetes. It includes the conditions of impaired fasting glucose (IFG) and impaired glucose tolerance (IGT) and diabetes mellitus (DM).	• Type II diabetes mellitus + sub-terms<br>• Impaired glucose tolerance + sub-terms
Obesity	HP:0001513	Accumulation of substantial excess body fat.	• All sub-terms
Hypertriglyceridemia	HP:0002155	An abnormal increase in the level of triglycerides in the blood	• All sub-terms
Intellectual disability	HP:0001249	Subnormal intellectual functioning which originates during the developmental period. Intellectual disability, previously referred to as mental retardation, has been defined as an IQ score below 70.	• All sub-terms
Neurodevelopmental delay	HP:0012758	None listed	• All sub-terms
Hypotonia	HP:0001252	Hypotonia is an abnormally low muscle tone (the amount of tension or resistance to movement in a muscle). Even when relaxed, muscles have a continuous and passive partial contraction which provides some resistance to passive stretching. Hypotonia thus manifests as diminished resistance to passive stretching. Hypotonia is not the same as muscle weakness, although the two conditions can co-exist.	• All sub-terms
Ataxia	HP:0001251	Cerebellar ataxia refers to ataxia due to dysfunction of the cerebellum. This causes a variety of elementary neurological deficits including asynergy (lack of coordination between muscles, limbs and joints), dysmetria (lack of ability to judge distances that can lead to under- or overshoot in grasping movements), and dysdiadochokinesia (inability to perform	• All sub-terms

		rapid movements requiring antagonizing muscle groups to be switched on and off repeatedly).	
Abnormality of brain morphology	HP:0012443	A structural abnormality of the brain, which has as its parts the forebrain, midbrain, and hindbrain.	• Abnormal brainstem morphology + sub-terms<br>• Abnormal cerebral ventricle morphology + sub-terms<br>• Abnormal midbrain morphology + sub-terms<br>• Abnormality of forebrain morphology + sub-terms<br>• Abnormality of hindbrain morphology + sub-terms
Polydactyly	HP:0010442	A congenital anomaly characterized by the presence of supernumerary fingers or toes.	• All sub-terms
Short stature	HP:0004322	A height below that which is expected according to age and gender norms. Although there is no universally accepted definition of short stature, many refer to "short stature" as height more than 2 standard deviations below the mean for age and gender (or below the 3rd percentile for age and gender dependent norms).	• All sub-terms
Thoracic hypoplasia	HP:0005257	None listed	• All sub-terms
Brachydactyly / micromelia	HP:0001156	Digits that appear disproportionately short compared to the hand/foot.	• All sub-terms
Micromelia	HP:0002983	The presence of abnormally small extremities.	• All sub-terms
Abnormality of dentition	HP:0000164	Any abnormality of the teeth	• All sub-terms
Abnormal oral morphology	HP:0031816	Any structural anomaly of the mouth, which is also known as the oral cavity.	• All sub-terms
OFD1-specific facial dysmorphic features	HP:0000316	Hypertelorism: Interpupillary distance more than 2 SD above the mean (alternatively, the appearance of an increased interpupillary distance or widely spaced eyes)	• This term only
	HP:0000430	Underdeveloped nasal alae: Thinned, deficient, or excessively arched ala nasi.	• This term only
	HP:0000347	Micrognathia: Developmental hypoplasia of the mandible.	• This term only

Supplementary Table 3: Participants reported solved or partially solved in GMC exit questionnaires with variants in ciliopathy genes of interest

RESEARCH ID	GMC exit report outcome	Reported Sex	100K Recruitment Category	Gene	Variant Zygosity	Consequence	HGVSc	HGVSp	GMC exit questionnaire ACMG Class
1	Solved	MALE	BBS	ALMS1	Het	FS	NM_015120.4:c.10775del	NP_055935.4:p.Thr3592LysfsTer6	Path
					Het	SG	NM_015120.4:c.11107C>T	NP_055935.4:p.Arg3703Ter	Path
2	Solved	FEMALE	CDS	ALMS1	Het	SG	NM_015120.4:c.10975C>T	NP_055935.4:p.Arg3659Ter	Path
					Het	SG; FS	NM_015120.4:c.4571dup	NP_055935.4:p.Tyr1524Ter	Path
3	Solved	MALE	RCD	ALMS1	Het	FS	NM_015120.4:c.284del	NP_055935.4:p.Pro95ArgfsTer19	Path
					Het	FS	NM_015120.4:c.1793del	NP_055935.4:p.Glu598GlyfsTer3	Path
4	Solved	FEMALE	LCA or EOSRD	ALMS1	Het	SG	NM_015120.4:c.10483C>T	NP_055935.4:p.Gln3495Ter	Path
					Het	FS	NM_015120.4:c.6590del	NP_055935.4:p.Lys2197SerfsTer10	Path
5	Solved	FEMALE	ID; RCD	ALMS1	Het	FS	NM_015120.4:c.6570del	NP_055935.4:p.Ser2191HisfsTer16	Path
					Het	FS	NM_015120.4:c.10831_10832del	NP_055935.4:p.Arg3611AlafsTer6	Path
6	Solved	MALE	BBS	ALMS1	Het	FS	NM_015120.4:c.11881dup	NP_055935.4:p.Ser3961PhefsTer12	Path
					Het	"Large delins"	Data missing	Data missing	Likely path
7	Solved	MALE	URUMD	ALMS1	Hom	FS	NM_015120.4:c.2515dup	NP_055935.4:p.Ser839PhefsTer8	Path
8	Solved	MALE	BBS	ALMS1	Hom	FS	NM_015120.4:c.4684_4690dup	NP_055935.4:p.Ile1564AsnfsTer20	Path
9	Solved	FEMALE	RCD	BBS1	Hom	Mis	NM_024649.5:c.1169T>G	NP_078925.3:p.Met390Arg	Path
10	Solved	FEMALE	RCD	BBS1	Hom	Mis	19)	NP_078925.3:p.Met390Arg	Path
11	Solved	FEMALE	RCD	BBS1	Hom	Mis	NM_024649.5:c.1169T>G	NP_078925.3:p.Met390Arg	Path
12	Solved	MALE	RCD	BBS1	Hom	Mis	NM_024649.5:c.1169T>G	NP_078925.3:p.Met390Arg	Path
13	Solved	FEMALE	RCD	BBS1	Hom	Mis	NM_024649.5:c.1169T>G	NP_078925.3:p.Met390Arg	Path
14	Solved	FEMALE	SEOO +/- OEF + SS	BBS1	Hom	Mis	NM_024649.5:c.1169T>G	NP_078925.3:p.Met390Arg	Path
15	Solved	FEMALE	ID	BBS1	Hom	Mis	NM_024649.5:c.1169T>G	NP_078925.3:p.Met390Arg	Path
16	Solved	MALE	BBS	BBS1	Hom	Mis	NM_024649.5:c.1169T>G	NP_078925.3:p.Met390Arg	Path
17	Solved	MALE	RCD	BBS1	Hom	Mis	NM_024649.5:c.1169T>G	NP_078925.3:p.Met390Arg	Path
18	Solved	MALE	CKD	BBS1	Hom	Mis	NM_024649.5:c.1169T>G	NP_078925.3:p.Met390Arg	Path
19	Partially	MALE	ID	BBS1	Het	Mis	NM_024649.5:c.1169T>G	NP_078925.3:p.Met390Arg	Path
					Het	SG	NM_024649.5:c.871C>T	NP_078925.3:p.Gln291Ter	Path
20	Solved	FEMALE	Mito D	BBS1	Hom	Mis	NM_024649.5:c.1169T>G	NP_078925.3:p.Met390Arg	Path
21	Partially	MALE	RDS	BBS10	Het	Mis	NM_024685.4:c.1230T>G	NP_078961.3:p.His410Gln	Likely path
					Het	FS	NM_024685.4:c.271dup	NP_078961.3:p.Cys91LeufsTer5	Path
22	Solved	MALE	CAKUT	CEP290	Het	FS	NM_025114.4:c.2848dup	NP_079390.3:p.Gln950ProfsTer6	Path
					Het	Mis	NM_025114.4:c.2817G>T	NP_079390.3:p.Lys939Asn	Likely path

23	Solved	FEMALE	JBTS	CEP290	Hom	SG	NM_025114.4:c.5932C>T	NP_079390.3:p.Arg1978Ter	Path
24	Solved	MALE	LCA or EOSRD	CEP290	Hom	In-frame deletion	NM_025114.4:c.4661_4663del	NP_079390.3:p.Glu1554del	Likely path
25	Solved	FEMALE	LCA or EOSRD	CEP290	Het	FS	NM_025114.4:c.5434_5435del	NP_079390.3:p.Glu1812LysfsTer5	Path
					Het	SG	NM_025114.4:c.5668G>T	NP_079390.3:p.Gly1890Ter	Path
26	Solved	FEMALE	CAKUT	CEP290	Hom	SG	NM_025114.4:c.4174G>T	NP_079390.3:p.Glu1392Ter	Likely path
27	Partially	MALE	ID	CEP290	Het	SG	NM_025114.4:c.322C>T	NP_079390.3:p.Arg108Ter	Path
					Het	FS	NM_025114.4:c.3422dup	NP_079390.3:p.Leu1141PhefsTer5	Path
28	Solved	MALE	RCD	CEP290	Het	SG	NM_025114.4:c.1984C>T	NP_079390.3:p.Gln662Ter	Path
					Het	SG	NM_025114.4:c.7048C>T	NP_079390.3:p.Gln2350Ter	Path
29	Solved	FEMALE	BBS	CEP290	Het	SG	NM_025114.4:c.5668G>T	NP_079390.3:p.Gly1890Ter	Path
					Het	SG	NM_025114.4:c.322C>T	NP_079390.3:p.Arg108Ter	Path
30	Solved	MALE	RCD	DYNC1H1	Hom	SG	NM_001080463.2:c.9836C>A	NP_001073932.1:p.Ser3279Ter	Path
31	Solved	MALE	USD	DYNC1H1	Het	Spl A	NM_001080463.2:c.10834-1G>A	-	Path
					Het	Spl Reg	NM_001080463.2:c.6140-5A>G	-	Likely path
32	Solved	MALE	RCD	OFD1	Hemi	FS	NM_003611.3:c.2680_2681del	NP_003602.1:p.Glu894ArgfsTer6	Path
33	Solved	FEMALE	RCD	NPHP1	Het	Mis	NM_001128178.3:c.1882C>T	NP_001121650.1:p.Arg628Trp	Likely path
					Het	"Whole gene deletion"	Data missing	Data missing	Not specified
34	Solved	MALE	UKFIYP	NPHP1	Hom	Mis	NM_001128178.3:c.859G>A	NP_001121650.1:p.Gly287Arg	Path
35	Solved	MALE	UKFIYP	NPHP1	Hom	SG	NM_001128178.3:c.1142G>A	NP_001121650.1:p.Trp381Ter	Path
36	Solved	FEMALE	UKFIYP	OFD1	Het	FS	NM_003611.3:c.1651_1654del	NP_003602.1:p.Thr551ProfsTer2	Path
37	Solved	FEMALE	SARMIRD	OFD1	Het	Mis	NM_003611.3:c.1363A>C	NP_003602.1:p.Lys455Gln	VUS
38	Solved	FEMALE	Craniosyn S	OFD1	Het	Spl Reg	NM_003611.3:c.382-4A>G	-	VUS
39	Solved	FEMALE	CKD	OFD1	Het	Spl A	NM_003611.3:c.112-1G>A	-	Path
40	Partially	FEMALE	RMCD	OFD1	Het	FS	NM_003611.3:c.306del	NP_003602.1:p.Glu103LysfsTer42	Likely path
41	Solved	MALE	CKD	TMEM67	Het	FS	NM_153704.6:c.103del	NP_714915.3:p.Gln35ArgfsTer52	Path
					Het	FS	NM_153704.6:c.415_416del	NP_714915.3:p.Asp139HisfsTer2	Path
42	Partially	MALE	ID	TMEM67	Het	Mis	NM_153704.6:c.1319G>A	NP_714915.3:p.Arg440Gln	Path
					Het	Mis	NM_153704.6:c.2498T>C	NP_714915.3:p.Ile833Thr	Likely path
43	Solved	MALE	RCD	CEP290	Het	FS	NM_025114.4:c.254dup	NP_079390.3:p.Asn85LysfsTer6	Likely path
44	Solved	MALE	LCA or EOSRD	CEP290	Hom	Mis	NM_025114.4:c.21G>T	NP_079390.3:p.Trp7Cys	Likely path

Abbreviations: 100K = 100,000 Genomes Project, GMC = Genomic Medicine Centre, ACMG = American College of Medical Genetics and Genomics, BBS = Bardet-Biedl syndrome, CDS = cone dysfunction syndrome, RCD = rod-cone dystrophy, LCA or EOSRD = Leber Congenital Amaurosis or Early-Onset Severe Retinal Dystrophy, ID = intellectual disability, URUMD = Ultra-rare undescribed monogenic disorders, SEOO +/- OEF + SS = Significant early-onset obesity with or without other endocrine features and short stature, CKD = cystic kidney disease, Mito D = mitochondrial disorders, RDS = rod-dysfunction syndrome, CAKUT = Congenital Anomaly of the Kidneys and Urinary Tract, JBTS = Joubert

syndrome, USD = Unexplained skeletal dysplasia, UKFIYP = Unexplained kidney failure in young people, SARMIRD = Single autosomal recessive mutation in rare disease, Craniosyn S = craniosynostosis syndromes, RMCD = Rare multisystem ciliopathy disorders, Het = heterozygous, Hom = homozygous, Hemi = hemizygous, FS = frameshift, SG = stop gain, Mis = missense, Spl A = splice acceptor, Spl Reg = splice region, Path = pathogenic, Likely path = likely pathogenic, VUS = variant of uncertain significance

Supplementary Table 4: Prioritised variants extracted through reverse phenotyping diagnostic research workflow

Step 2 workflow inputs and outputs: filtering and prioritisation of SNVs using custom Python script

INPUTS

INPUT SNV DATA: All SNVs from the 100K dataset for each selected ciliopathy gene generated by Gene-Variant Workflow. Separate lists for participants called on GrCh37 and GrCh38

Gene	ALMS1		BBS1		BBS10		DYNC2H1		WDR34		OFD1		NPHP1		TMEM67		CEP290	
Build	GrCh37	GrCh38	GrCh37	GrCh38	GrCh37	GrCh38	GrCh37	GrCh38	GrCh37	GrCh38	GrCh37	GrCh38	GrCh37	GrCh38	GrCh37	GrCh38	GrCh37	GrCh38
# un-filtered Gene-Variant Workflow variants	52420	287121	24050	71969	166	601	80615	284569	7636	234958	2122	27257	30997	104051	28384	95596	19436	96000

PROCESS: filter using custom python script filter_gene_variant_workflow.py
A: Exclude common variants: 100K MAF ≥ 0.002; gnomAD AF ≥ 0.002
B: Exclude variants called in non-canonical transcripts
⇓

Gene	ALMS1		BBS1		BBS10		DYNC2H1		WDR34		OFD1		NPHP1		TMEM67		CEP290	
# filtered variants: rare, canonical transcripts only	11862	43098	1217	3802	153	588	16127	59165	1465	4939	279	4365	3399	12254	2810	10226	3740	14200

PROCESS: extract prioritised SNV sub-lists using custom python script filter_gene_variant_workflow.py:

- ClinVar pathogenic/likely pathogenic
- VEP High Impact (stop_gained, stop_lost, start_lost, splice_acceptor_variant, splice_donor_variant, frameshift_variant, transcript_ablation, transcript_amplification)
- SIFT deleterious missense

OUTPUTS

Gene	ALMS1		BBS1		BBS10		DYNC2H1		WDR34		OFD1		NPHP1		TMEM67		CEP290	
Total ClinVar Pathogenic	13	43	1	14	5	22	16	58	2	9	0	64	3	8	10	36	22	78
Total VEP High Impact	30	130	2	22	5	28	19	141	4	38	0	70	7	35	11	57	36	167
Total SIFT deleterious missense	167	643	33	86	18	86	125	556	32	107	5	75	26	79	33	167	84	344

DISTRIBUTION OF PRIORITISED VARIANTS BETWEEN DIFFERENT PRIORITISED SNV SUB-LISTS																		
Gene	ALMS1		BBS1		BBS10		DYNC2H1		WDR34		OFD1		NPHP1		TMEM67		CEP290	
# ClinVar Pathogenic + VEP High Impact	13	43	0	11	5	17	5	26	1	6	0	58	2	7	4	20	19	73
# ClinVar pathogenic + SIFT deleterious missense	0	0	1	3	0	5	10	30	1	3	0	5	1	1	6	14	2	4
# VEP High Impact (only)	17	87	2	11	0	11	13	115	3	32	0	12	5	28	7	37	17	94
# SIFT deleterious missense (only)	167	643	32	83	18	81	115	526	31	104	5	70	25	78	27	153	82	340
# ClinVar Pathogenic (only)	0	0	0	0	0	0	1	2	0	0	0	1	0	0	0	2	1	1
Total	197	773	35	108	23	114	144	699	36	145	5	146	33	114	44	226	121	512

Step 3 workflow inputs and outputs: search for potentially pathogenic SVs using SVRare script

INPUTS

INPUT DATA: PlateKey identifiers for all unsolved 100K participants (probands and affected relatives) with heterozygous ClinVar pathogenic or VEP high impact prioritised SNVs in one of the nine ciliopathy genes
N = 801 participants

PROCESS: Submitted to SVRare script (Yu et al, 2021)
Extracts participants with SVs called by Manta and/or Canvas with ≤ 10 calls across the 100K database, overlapping coding regions of the 9 ciliopathy genes
⇓

OUTPUTS

Gene	ALMS1		BBS1		BBS10		DYNC2H1		WDR34		OFD1		NPHP1		TMEM67		CEP290	
# Prioritised SNVs	0	1	0	0	0	0	1	0	0	0	0	1	0	0	0	0	0	1
Impression	N/a	LP	N/a	N/a	N/a	N/a	LP	N/a	N/a	N/a	N/a	Excl: 2nd hit in different gene	N/a	N/a	N/a	N/a	N/a	Excl: alternative diagnosis

Step 4 workflow inputs and outputs: search for novel splicing variants using custom SpliceAI script

INPUTS

INPUT DATA: all rare variants (100K MAF ≤ 0.002; gnomAD AF ≤ 0.002) called in canonical transcripts in the nine ciliopathy genes identified in unsolved 100K participants
AS PER Step 2: Gene-Variant Workflow rare SNVs called in canonical transcripts filtered through custom python script (filter_gene_variant_workflow.py)

PROCESS: Run through custom SpliceAI Python script (find_variants_by_gene_and_SpliceAI_score.py)

⇓

FILTERING:

- Variants called in unaffected relatives excluded
- Variants with SpliceAI delta score (DS) > 0.5 retained
- Variants already assessed on other SNV prioritised sub-lists excluded

⇓

OUTPUTS

Gene	ALMS1		BBS1		BBS10		DYNC2H1		WDR34		OFD1		NPHP1		TMEM67		CEP290	
# rare variants with SpliceAI DS >0.5	1	22	3	10	0	1	7	53	1	9	0	10	3	12	2	15	4	34

The number of variants input, filtered and prioritised in steps 2, 3 and 4 of the reverse phenotyping diagnostic research workflow. Note that 100K participants had genomes called on GrCh37 or GrCh38 depending on when they were recruited to the project.
Abbreviations: SNV = single nucleotide variant, 100K = 100,000 Genomes Project, AF = allele frequency, MAF = maximum allele frequency, VEP = Variant Effect Predictor, SV = structural variant, Excl = excluded

Supplementary Data 1: Duplex PCR assay of a *BBS1* exon 13 mobile element insertion

The patient presented with congenital right ptosis, childhood onset high myopia, rod/cone dysfunction, autism, dyspraxia and postaxial polydactyly on the left hand and foot that were removed in childhood. The patient was recruited to the 100,000 Genomes Project (100K) for whole genome sequencing, following identification of a heterozygous pathogenic variant in an autosomal recessive disease gene through mainstream testing. The *BBS1* missense mutation, NM_024649.5:c.1169T>G, NP_078925.3:p.(Met390Arg), was insufficient to confirm the diagnosis in the absence of a second pathogenic variant. 100K tiering failed to identify a second deleterious allele in *BBS1*. Manual inspection of the aligned sequence reads using the Integrative Genome Browser (IGV) v.2.4.10 (http://software.broadinstitute.org/software/igv/) (33) and interrogation of soft-clipped reads using BLAT (http://genome.ucsc.edu/cgi-bin/hgBlat) (34), revealed a soft-clipped read signature that was consistent with a 2.4 kb insertion of an SVA F family element mobile element (35).

To confirm the *BBS1* heterozygous missense variant, c.1169T>C, a PCR amplicon was first optimised; each reaction comprised 0.5 µL of genomic DNA (~50 ng/µL) 19.3 µL MegaMix PCR reagent (Microzone Ltd., Haywards Heath, UK) and 0.1 µL each of 10 µM forward (d<u>TGTAAAACGACGGCCAGT</u>AAAGGCAGCATTGTGAAGGG) and reverse (d<u>CAGGAAACAGCTATGACC</u>CCCTTCACTCCCGACTTCAA) primers. Thermocycling conditions comprised 94°C for 5 minutes then 30 cycles of 94°C for 30 seconds, 55°C for 1 minute and 72°C for 2 minutes before a final extension step at 72°C for 5 minutes. Amplification products were resolved on a 1% Tris-borate-EDTA agarose gel, before being extracted and purified using a QIAquick column (Qiagen GmbH, Hilden, Germany), then Sanger sequenced using an ABI3730 following manufacturer’s protocols throughout (Life Technologies Ltd., Paisley, UK). Sequence chromatograms were analysed using 4Peaks v.1.8 (http://nucleobytes.com/4peaks/index.html). Universal sequence tags (underlined) were incorporated into primer tails for use with our routine diagnostic workflow.

To verify the apparent *BBS1* exon 13 mobile element insertion, we implemented the duplex PCR assay as described previously (35). Each reaction comprised 0.5 µL of genomic DNA (~50 ng/µL) 19.2 µL of MegaMix PCR reagent and 0.1 µL each of 10 µM primer. These included a common intron 12 forward (dCACAGTACTCCACAAATAACTGCT), an intron 13 reverse

(dATTCCCCCAGCTTTGCTGT) and insertion-specific reverse (dCAGCCTGGGCACCATTGA) primer. Thermocycling conditions required 35 cycles, but were otherwise as described above. Amplification products specific for the normal (440 bp) and insertion-containing (270 bp) allele were resolved on a 2% TRIS-borate-EDTA agarose gel prior to gel extraction and Sanger sequencing. To determine the precise sequence of the downstream target site duplication a further PCR was optimised for Sanger sequencing, using previously reported forward (F9: dAGTACCCAGGGACAAACACT) and reverse (R5: dGTCTTTCGGGGCACATTGAG) primers (35). Analysis of parental alignments supported the mobile element insertion being in *trans* with the maternally-inherited c.1169T>C mutation, with Sanger sequencing confirming the presence of the insertion in the proband and his father.

Supplementary references

1. Niederlova V, Modrak M, Tsyklauri O, Huranova M, Stepanek O. Meta-analysis of genotype-phenotype associations in Bardet-Biedl syndrome uncovers differences among causative genes. Hum Mutat. 2019;40(11):2068-87.
2. Shamseldin HE, Shaheen R, Ewida N, Bubshait DK, Alkuraya H, Almardawi E, Howaidi A, Sabr Y, Abdalla EM, Alfaifi AY, Alghamdi JM, Alsagheir A, Alfares A, Morsy H, Hussein MH, Al-Muhaizea MA, Shagrani M, Al Sabban E, Salih MA, Meriki N, Khan R, Almugbel M, Qari A, Tulba M, Mahnashi M, Alhazmi K, Alsalamah AK, Nowilaty SR, Alhashem A, Hashem M, Abdulwahab F, Ibrahim N, Alshidi T, AlObeid E, Alenazi MM, Alzaidan H, Rahbeeni Z, Al-Owain M, Sogaty S, Seidahmed MZ, Alkuraya FS. The morbid genome of ciliopathies: an update. Genet Med. 2020;22(6):1051-60.
3. Forsyth R, Gunay-Aygun M. Bardet-Biedl Syndrome Overview. In: Adam MP, Ardinger HH, Pagon RA, Wallace SE, Bean LJH, Mirzaa G, et al., editors. GeneReviews(®). Seattle (WA): University of Washington, Seattle. Copyright © 1993-2021, University of Washington, Seattle. GeneReviews is a registered trademark of the University of Washington, Seattle. All rights reserved.; 1993.
4. Paisey RB, Steeds R, Barrett T, Williams D, Geberhiwot T, Gunay-Aygun M. Alström Syndrome. In: Adam MP, Ardinger HH, Pagon RA, Wallace SE, Bean LJH, Mirzaa G, et al., editors. GeneReviews(®). Seattle (WA): University of Washington, Seattle. Copyright © 1993-2021, University of Washington, Seattle. GeneReviews is a registered trademark of the University of Washington, Seattle. All rights reserved.; 1993.
5. Aldrees A, Abdelkader E, Al-Habboubi H, Alrwebah H, Rahbeeni Z, Schatz P. Non-syndromic retinal dystrophy associated with homozygous mutations in the ALMS1 gene. Ophthalmic Genet. 2019;40(1):77-9.
6. Hull S, Kiray G, Chiang JP, Vincent AL. Molecular and phenotypic investigation of a New Zealand cohort of childhood-onset retinal dystrophy. Am J Med Genet C Semin Med Genet. 2020;184(3):708-17.
7. Lazar CH, Kimchi A, Namburi P, Mutsuddi M, Zelinger L, Beryozkin A, Ben-Simhon S, Obolensky A, Ben-Neriah Z, Argov Z, Pikarsky E, Fellig Y, Marks-Ohana D, Ratnapriya R, Banin E, Sharon D, Swaroop A. Nonsyndromic Early-Onset Cone-Rod Dystrophy and Limb-Girdle Muscular Dystrophy in a Consanguineous Israeli Family are Caused by Two Independent yet Linked Mutations in ALMS1 and DYSF. Hum Mutat. 2015;36(9):836-41.
8. Louw JJ, Corveleyn A, Jia Y, Iqbal S, Boshoff D, Gewillig M, Peeters H, Moerman P, Devriendt K. Homozygous loss-of-function mutation in ALMS1 causes the lethal disorder mitogenic cardiomyopathy in two siblings. Eur J Med Genet. 2014;57(9):532-5.
9. Bachmann-Gagescu R, Dempsey JC, Phelps IG, O'Roak BJ, Knutzen DM, Rue TC, Ishak GE, Isabella CR, Gorden N, Adkins J, Boyle EA, de Lacy N, O'Day D, Alswaid A, Ramadevi AR, Lingappa L, Lourenco C, Martorell L, Garcia-Cazorla A, Ozyurek H, Haliloglu G, Tuysuz B, Topcu M, Chance P, Parisi MA, Glass IA, Shendure J, Doherty D. Joubert syndrome: a model for untangling recessive disorders with extreme genetic heterogeneity. J Med Genet. 2015;52(8):514-22.
10. Vilboux T, Doherty DA, Glass IA, Parisi MA, Phelps IG, Cullinane AR, Zein W, Brooks BP, Heller T, Soldatos A, Oden NL, Yildirimli D, Vemulapalli M, Mullikin JC, Nisc Comparative Sequencing P, Malicdan MCV, Gahl WA, Gunay-Aygun M. Molecular genetic findings and

clinical correlations in 100 patients with Joubert syndrome and related disorders prospectively evaluated at a single center. Genet Med. 2017;19(8):875-82.

11. Parisi M, Glass I. Joubert Syndrome. In: Adam MP, Ardinger HH, Pagon RA, Wallace SE, Bean LJH, Mirzaa G, et al., editors. GeneReviews(®). Seattle (WA): University of Washington, Seattle. Copyright © 1993-2021, University of Washington, Seattle. GeneReviews is a registered trademark of the University of Washington, Seattle. All rights reserved.; 1993.
12. Suzuki T, Miyake N, Tsurusaki Y, Okamoto N, Alkindy A, Inaba A, Sato M, Ito S, Muramatsu K, Kimura S, Ieda D, Saitoh S, Hiyane M, Suzumura H, Yagyu K, Shiraishi H, Nakajima M, Fueki N, Habata Y, Ueda Y, Komatsu Y, Yan K, Shimoda K, Shitara Y, Mizuno S, Ichinomiya K, Sameshima K, Tsuyusaki Y, Kurosawa K, Sakai Y, Haginoya K, Kobayashi Y, Yoshizawa C, Hisano M, Nakashima M, Saitsu H, Takeda S, Matsumoto N. Molecular genetic analysis of 30 families with Joubert syndrome. Clin Genet. 2016;90(6):526-35.
13. Otto EA, Tory K, Attanasio M, Zhou W, Chaki M, Paruchuri Y, Wise EL, Wolf MT, Utsch B, Becker C, Nurnberg G, Nurnberg P, Nayir A, Saunier S, Antignac C, Hildebrandt F. Hypomorphic mutations in meckelin (MKS3/TMEM67) cause nephronophthisis with liver fibrosis (NPHP11). J Med Genet. 2009;46(10):663-70.
14. Hartill V, Szymanska K, Sharif SM, Wheway G, Johnson CA. Meckel-Gruber Syndrome: An Update on Diagnosis, Clinical Management, and Research Advances. Front Pediatr. 2017;5(244).
15. Iannicelli M, Brancati F, Mougou-Zerelli S, Mazzotta A, Thomas S, Elkhartoufi N, Travaglini L, Gomes C, Ardissino GL, Bertini E, Boltshauser E, Castorina P, D'Arrigo S, Fischetto R, Leroy B, Loget P, Bonniere M, Starck L, Tantau J, Gentilin B, Majore S, Swistun D, Flori E, Lalatta F, Pantaleoni C, Penzien J, Grammatico P, Dallapiccola B, Gleeson JG, Attie-Bitach T, Valente EM. Novel TMEM67 mutations and genotype-phenotype correlates in meckelin-related ciliopathies. Hum Mutat. 2010;31(5):E1319-31.
16. Brancati F, Iannicelli M, Travaglini L, Mazzotta A, Bertini E, Boltshauser E, D'Arrigo S, Emma F, Fazzi E, Gallizzi R, Gentile M, Loncarevic D, Mejaski-Bosnjak V, Pantaleoni C, Rigoli L, Salpietro CD, Signorini S, Stringini GR, Verloes A, Zabloka D, Dallapiccola B, Gleeson JG, Valente EM. MKS3/TMEM67 mutations are a major cause of COACH Syndrome, a Joubert Syndrome related disorder with liver involvement. Hum Mutat. 2009;30(2):E432-42.
17. Doherty D, Parisi MA, Finn LS, Gunay-Aygun M, Al-Mateen M, Bates D, Clericuzio C, Demir H, Dorschner M, van Essen AJ, Gahl WA, Gentile M, Gorden NT, Hikida A, Knutzen D, Ozyurek H, Phelps I, Rosenthal P, Verloes A, Weigand H, Chance PF, Dobyns WB, Glass IA. Mutations in 3 genes (MKS3, CC2D2A and RPGRIP1L) cause COACH syndrome (Joubert syndrome with congenital hepatic fibrosis). J Med Genet. 2010;47(1):8-21.
18. Travaglini L, Brancati F, Attie-Bitach T, Audollent S, Bertini E, Kaplan J, Perrault I, Iannicelli M, Mancuso B, Rigoli L, Rozet JM, Swistun D, Tolentino J, Dallapiccola B, Gleeson JG, Valente EM, Zankl A, Leventer R, Grattan-Smith P, Janecke A, D'Hooghe M, Sznajer Y, Van Coster R, Demerleir L, Dias K, Moco C, Moreira A, Kim CA, Maegawa G, Petkovic D, Abdel-Salam GM, Abdel-Aleem A, Zaki MS, Marti I, Quijano-Roy S, Sigaudy S, de Lonlay P, Romano S, Touraine R, Koenig M, Lagier-Tourenne C, Messer J, Collignon P, Wolf N, Philippi H, Kitsiou Tzeli S, Halldorsson S, Johannsdottir J, Ludvigsson P, Phadke SR, Udani V, Stuart B, Magee A, Lev D, Michelson M, Ben-Zeev B, Fischetto R, Benedicenti F, Stanzial F, Borgatti R, Accorsi P, Battaglia S, Fazzi E, Giordano L, Pinelli L, Boccone L, Bigoni S, Ferlini A, Donati MA, Caridi G, Divizia MT, Faravelli F, Ghiggeri G, Pessagno A, Briguglio M,

Briuglia S, Salpietro CD, Tortorella G, Adami A, Castorina P, Lalatta F, Marra G, Riva D, Scelsa B, Spaccini L, Uziel G, Del Giudice E, Laverda AM, Ludwig K, Permunian A, Suppiej A, Signorini S, Uggetti C, Battini R, Di Giacomo M, Cilio MR, Di Sabato ML, Leuzzi V, Parisi P, Pollazzon M, Silengo M, De Vescovi R, Greco D, Romano C, Cazzagon M, Simonati A, Al-Tawari AA, Bastaki L, Mégarbané A, Sabolic Avramovska V, de Jong MM, Stromme P, Koul R, Rajab A, Azam M, Barbot C, Martorell Sampol L, Rodriguez B, Pascual-Castroviejo I, Teber S, Anlar B, Comu S, Karaca E, Kayserili H, Yüksel A, Akcakus M, Al Gazali L, Sztriha L, Nicholl D, Woods CG, Bennett C, Hurst J, Sheridan E, Barnicoat A, Hennekam R, Lees M, Blair E, Bernes S, Sanchez H, Clark AE, DeMarco E, Donahue C, Sherr E, Hahn J, Sanger TD, Gallager TE, Dobyns WB, Daugherty C, Krishnamoorthy KS, Sarco D, Walsh CA, McKanna T, Milisa J, Chung WK, De Vivo DC, Raynes H, Schubert R, Seward A, Brooks DG, Goldstein A, Caldwell J, Finsecke E, Maria BL, Holden K, Cruse RP, Swoboda KJ, Viskochil D. Expanding CEP290 mutational spectrum in ciliopathies. Am J Med Genet A. 2009;149a(10):2173-80.

19. Kumaran N, Pennesi ME, Yang P, Trzupek KM, Schlechter C, Moore AT, Weleber RG, Michaelides M. Leber Congenital Amaurosis / Early-Onset Severe Retinal Dystrophy Overview. In: Adam MP, Ardinger HH, Pagon RA, Wallace SE, Bean LJH, Mirzaa G, et al., editors. GeneReviews(®). Seattle (WA): University of Washington, Seattle. Copyright © 1993-2021, University of Washington, Seattle. GeneReviews is a registered trademark of the University of Washington, Seattle. All rights reserved.; 1993.
20. Coppieters F, Lefever S, Leroy BP, De Baere E. *CEP290*, a gene with many faces: mutation overview and presentation of *CEP290*. Human Mutation. 2010;31(10):1097-108.
21. Adams M, Simms RJ, Abdelhamed Z, Dawe HR, Szymanska K, Logan CV, Wheway G, Pitt E, Gull K, Knowles MA, Blair E, Cross SH, Sayer JA, Johnson CA. A meckelin-filamin A interaction mediates ciliogenesis. Human molecular genetics. 2012;21(6):1272-86.
22. Chang B, Khanna H, Hawes N, Jimeno D, He S, Lillo C, Parapuram SK, Cheng H, Scott A, Hurd RE, Sayer JA, Otto EA, Attanasio M, O'Toole JF, Jin G, Shou C, Hildebrandt F, Williams DS, Heckenlively JR, Swaroop A. In-frame deletion in a novel centrosomal/ciliary protein CEP290/NPHP6 perturbs its interaction with RPGR and results in early-onset retinal degeneration in the rd16 mouse. Hum Mol Genet. 2006;15(11):1847-57.
23. Leitch CC, Zaghloul NA, Davis EE, Stoetzel C, Diaz-Font A, Rix S, Alfadhel M, Lewis RA, Eyaid W, Banin E, Dollfus H, Beales PL, Badano JL, Katsanis N. Hypomorphic mutations in syndromic encephalocele genes are associated with Bardet-Biedl syndrome. Nature Genetics. 2008;40:443.
24. Brancati F, Camerota L, Colao E, Vega-Warner V, Zhao X, Zhang R, Bottillo I, Castori M, Caglioti A, Sangiuolo F, Novelli G, Perrotti N, Otto EA. Biallelic variants in the ciliary gene TMEM67 cause RHYNS syndrome. European journal of human genetics : EJHG. 2018;26(9):1266-71.
25. Schmidts M, Arts HH, Bongers EM, Yap Z, Oud MM, Antony D, Duijkers L, Emes RD, Stalker J, Yntema JB, Plagnol V, Hoischen A, Gilissen C, Forsythe E, Lausch E, Veltman JA, Roeleveld N, Superti-Furga A, Kutkowska-Kazmierczak A, Kamsteeg EJ, Elçioğlu N, van Maarle MC, Graul-Neumann LM, Devriendt K, Smithson SF, Wellesley D, Verbeek NE, Hennekam RC, Kayserili H, Scambler PJ, Beales PL, Knoers NV, Roepman R, Mitchison HM. Exome sequencing identifies DYNC2H1 mutations as a common cause of asphyxiating thoracic dystrophy (Jeune syndrome) without major polydactyly, renal or retinal involvement. J Med Genet. 2013;50(5):309-23.
26. Baujat G, Huber C, El Hokayem J, Caumes R, Do Ngoc Thanh C, David A, Delezoide AL, Dieux-Coeslier A, Estournet B, Francannet C, Kayirangwa H, Lacaille F, Le Bourgeois M,

Martinovic J, Salomon R, Sigaudy S, Malan V, Munnich A, Le Merrer M, Le Quan Sang KH, Cormier-Daire V. Asphyxiating thoracic dysplasia: clinical and molecular review of 39 families. J Med Genet. 2013;50(2):91-8.
27. Huber C, Wu S, Kim AS, Sigaudy S, Sarukhanov A, Serre V, Baujat G, Le Quan Sang KH, Rimoin DL, Cohn DH, Munnich A, Krakow D, Cormier-Daire V. WDR34 mutations that cause short-rib polydactyly syndrome type III/severe asphyxiating thoracic dysplasia reveal a role for the NF-κB pathway in cilia. Am J Hum Genet. 2013;93(5):926-31.
28. Schmidts M, Vodopiutz J, Christou-Savina S, Cortés CR, McInerney-Leo AM, Emes RD, Arts HH, Tüysüz B, D'Silva J, Leo PJ, Giles TC, Oud MM, Harris JA, Koopmans M, Marshall M, Elçioglu N, Kuechler A, Bockenhauer D, Moore AT, Wilson LC, Janecke AR, Hurles ME, Emmet W, Gardiner B, Streubel B, Dopita B, Zankl A, Kayserili H, Scambler PJ, Brown MA, Beales PL, Wicking C, Duncan EL, Mitchison HM. Mutations in the gene encoding IFT dynein complex component WDR34 cause Jeune asphyxiating thoracic dystrophy. Am J Hum Genet. 2013;93(5):932-44.
29. Halbritter J, Porath JD, Diaz KA, Braun DA, Kohl S, Chaki M, Allen SJ, Soliman NA, Hildebrandt F, Otto EA. Identification of 99 novel mutations in a worldwide cohort of 1,056 patients with a nephronophthisis-related ciliopathy. Hum Genet. 2013;132(8):865-84.
30. Stokman M, Lilien M, Knoers N. Nephronophthisis. In: Adam MP, Ardinger HH, Pagon RA, Wallace SE, Bean LJH, Mirzaa G, et al., editors. GeneReviews(®). Seattle (WA): University of Washington, Seattle. Copyright © 1993-2021, University of Washington, Seattle. GeneReviews is a registered trademark of the University of Washington, Seattle. All rights reserved.; 1993.
31. Parisi MA, Bennett CL, Eckert ML, Dobyns WB, Gleeson JG, Shaw DW, McDonald R, Eddy A, Chance PF, Glass IA. The NPHP1 gene deletion associated with juvenile nephronophthisis is present in a subset of individuals with Joubert syndrome. Am J Hum Genet. 2004;75(1):82-91.
32. Bruel AL, Franco B, Duffourd Y, Thevenon J, Jego L, Lopez E, Deleuze JF, Doummar D, Giles RH, Johnson CA, Huynen MA, Chevrier V, Burglen L, Morleo M, Desguerres I, Pierquin G, Doray B, Gilbert-Dussardier B, Reversade B, Steichen-Gersdorf E, Baumann C, Panigrahi I, Fargeot-Espaliat A, Dieux A, David A, Goldenberg A, Bongers E, Gaillard D, Argente J, Aral B, Gigot N, St-Onge J, Birnbaum D, Phadke SR, Cormier-Daire V, Eguether T, Pazour GJ, Herranz-Pérez V, Goldstein JS, Pasquier L, Loget P, Saunier S, Mégarbané A, Rosnet O, Leroux MR, Wallingford JB, Blacque OE, Nachury MV, Attie-Bitach T, Rivière JB, Faivre L, Thauvin-Robinet C. Fifteen years of research on oral-facial-digital syndromes: from 1 to 16 causal genes. J Med Genet. 2017;54(6):371-80.
33. Thorvaldsdóttir H, Robinson JT, Mesirov JP. Integrative Genomics Viewer (IGV): high-performance genomics data visualiza- tion and exploration. Brief Bioinform. 2013;14:178-92.
34. Kent WJ. BLAT-the BLAST-like Alignment Tool. Genome Res. 2002;12:656-64.
35. Delvallée C, Nicaise S, Antin M, Leuvrey AS, Nourisson E, Leitch CC, et al. A BBS1 SVA F retrotransposon insertion is a frequent cause of Bardet-Biedl syndrome. Clin Genet. 2021;99:318-324.
